# Origin of biological homochirality by crystallization of an RNA precursor on a magnetic surface

**DOI:** 10.1126/sciadv.adg8274

**Published:** 2023-06-07

**Authors:** S. Furkan Ozturk, Ziwei Liu, John D. Sutherland, Dimitar D. Sasselov

**Affiliations:** ^1^ Department of Physics, Harvard University, Cambridge, MA 02138, USA.; ^2^ MRC Laboratory of Molecular Biology, Cambridge Biomedical Campus, Cambridge CB2 0QH, UK.; ^3^ Department of Astronomy, Harvard University, Cambridge, MA 02138, USA.

## Abstract

Homochirality is a signature of life on Earth, yet its origins remain an unsolved puzzle. Achieving homochirality is essential for a high-yielding prebiotic network capable of producing functional polymers like RNA and peptides on a persistent basis. Because of the chiral-induced spin selectivity effect, which established a strong coupling between electron spin and molecular chirality, magnetic surfaces can act as chiral agents and be templates for the enantioselective crystallization of chiral molecules. Here, we studied the spin-selective crystallization of racemic ribo-aminooxazoline (RAO), an RNA precursor, on magnetite (Fe_3_O_4_) surfaces, achieving an unprecedented enantiomeric excess (ee) of about 60%. Following the initial enrichment, we then obtained homochiral (100% ee) crystals of RAO after a subsequent crystallization. Our results demonstrate a prebiotically plausible way of achieving system-level homochirality from completely racemic starting materials, in a shallow-lake environment on early Earth where sedimentary magnetite deposits are expected to be common.

## Teaser

Homochirality was achieved by the spin-selective crystallization of a racemic RNA precursor on a magnetite surface.

## INTRODUCTION

Understanding the origins of biomolecular homochirality is essential for understanding the origins of life, and the origin of homochirality remains a long-standing mystery since Pasteur (*1*) found the molecular asymmetry of organic compounds in 1848. Achieving a homochiral state early in the prebiotic synthesis of the monomers would be very beneficial for successful polymerization and the overall robustness of the entire synthetic network (*2*, *3*). The prebiotic need for high yields combined with high selectivity requires a persistent and well-matched pair of a chiral symmetry-breaking agent and an amplification mechanism.

Multiple studies [see (*4*, *5*) for review] have identified ways to induce an initial chiral imbalance, by a symmetry-breaking agent, like parity-violating energy difference (*6*), selective adsorption on surfaces (*7*), conglomerate resolution (*8*, *9*), circularly polarized light (*10*), and longitudinally spin-polarized electrons and muons (e.g., in cosmic ray showers) (*11*, *12*). Others have focused on the amplification of such initial imbalance to reach homochirality [see (*13*, *14*) for review]. A few studies (e.g., Viedma ripening) have realized a spontaneous symmetry breaking in racemizing compounds, followed by amplification by means of conglomerate crystallization to reach homochirality (*15*, *16*). Blackmond and colleagues realized that reaching homochirality in a single compound per se is not sufficient to reach system-level homochirality and studied the interchange of enantiomeric excess (ee) between amino acids and sugars (*17*–*19*). However, no studies have shown a way to pair a nondestructive chiral agent with a chiral amplification scheme to achieve a persistent, system-level homochiral state, especially in prebiotically relevant chemistry.

One of the central molecules for prebiotic synthetic networks emerged in 1970 from the work of Sanchez and Orgel (*20*), who identified the aminooxazolines (ribo, arabino, xylo, and lyxo) as organic intermediates useful for the synthesis of a wide variety of nucleotides, the monomers of nucleic acids (RNA and DNA). Later studies by Sutherland’s group (*21*, *22*) culminated in the prebiotic synthesis of pyrimidine nucleotides (*23*) with aminooxazolines as the precursors and the consequent development of an entire synthetic network for nucleotides and amino acids (*24*). Follow-up studies highlighted ribo-aminooxazoline (RAO) as a key compound to resolve the origin of homochirality due to its centrality in the synthesis of nucleotides and crystallization properties (*22*, *25*–*27*). Hein *et al.* (*17*) further emphasized the importance of RAO and obtained enantiopure RAO crystals directly from glyceraldehyde by kinetic resolution of the latter with enantioenriched proline. However, the search for a prebiotically plausible mechanism that can both induce and amplify a chiral bias in an RNA precursor from a fully racemic starting material has remained to be an open problem.

Biological systems comprise many homochiral molecules. In principle, such a state could be achieved by separately resolving each individual chiral compound. However, a more attractive solution would be to establish the homochirality in a compound from which propagation of homochirality to the whole system could occur. Emergence of homochirality at the stage of RAO sets the stage for the propagation of homochirality through RNA to peptides and thence through enantioselective catalysis to metabolites.

In our recent work, we proposed a symmetry-breaking agent that can trigger such an amplification in the reduction reactions, leading up to the production of the chiral 3-carbon sugar glyceraldahyde (itself the precursor to RAO). Our proposed mechanism uses the strong coupling between the electron spin and molecular chirality as established by the chiral-induced spin selectivity (CISS) effect (*28*). We identified evaporative lakes with authigenic iron-oxide sediments as prebiotically plausible settings (*29*), in which enantioselective reduction reactions can occur close to the magnetized surface by spin-polarized photoelectrons released from the magnetite by solar ultraviolet (UV) light.

**Fig. 1.**
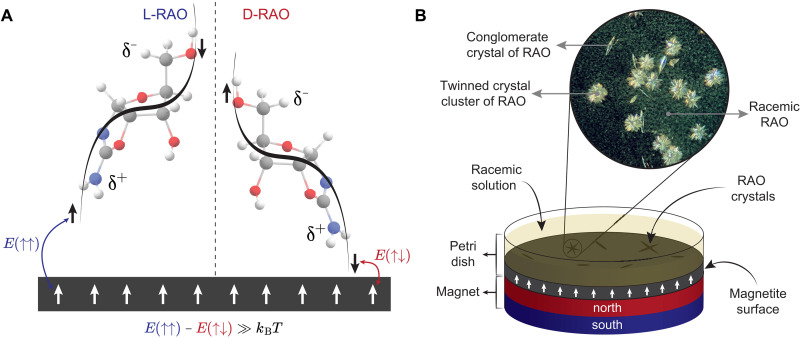
The mechanism of spin-selective crystallization due to the CISS effect and the experimental setup. (**A**) As molecules approach a surface, they transiently acquire an induced charge polarization. Because of the CISS effect, transient charge polarization of a chiral molecule is accompanied by spin polarization. The spin state associated with the charge poles is determined by the handedness of the chiral molecule. Because the magnetic surface itself is spin-polarized, it kinetically favors (akin to a seed crystal) the enantiomer whose transient spin state results in a lower-energy spin-exchange interaction. The lower-energy overlap with the magnetic surface is singlet-like (red, ↑↓) and the higher-energy overlap is triplet-like (blue, ↑↑). The energy difference between these two configurations is higher than the room temperature, *k*_B_*T*; therefore, the effect robustly manifests itself. (**B**) Schematic of the setup used in the crystallization experiments and a sample microscope image of the RAO crystals on a magnetite surface from a direct crystallization experiment. The image shows the magnetite surface as the black background and the needle-shaped conglomerate crystals of RAO formed on the surface, as well as the twinned crystals with stochastically arranged needles of D- and L-RAO, and racemic RAO in the form of a flaky powder suspended in the water column above the surface.

**Fig. 2.**
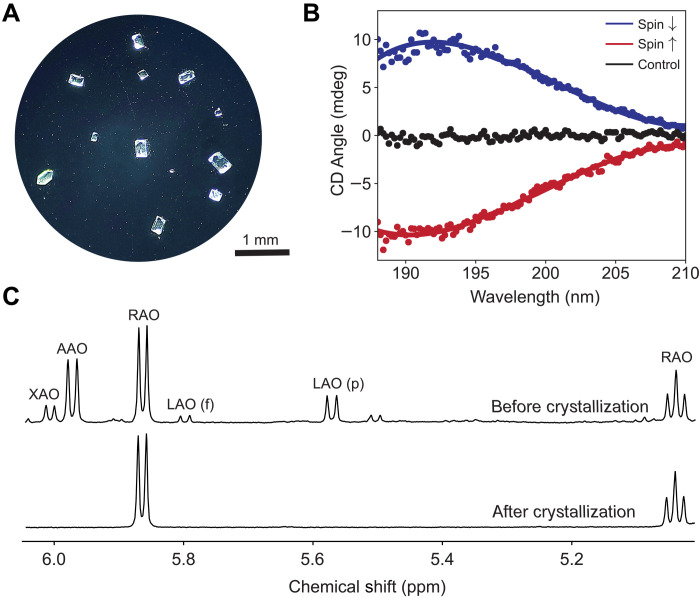
Stereoselective and enantioselective crystallization of RAO. (**A**) A microscope image of the nearly enantiopure RAO crystals formed on a magnetite surface from their racemic solution. (**B**) Circular dichroism (CD) spectra of the crystals formed on the magnetite surface. The red (blue) spectrum corresponds to D-RAO (L-RAO) crystals formed when the magnetite surface is magnetized parallel (antiparallel) to the surface normal. Both spectra are obtained when racemic RAO is recrystallized, and they show a cumulative ee of about 60% for the whole surface. The black spectrum corresponds to crystallization on a nonmagnetic, silicon surface in the presence of a magnetic field, and it shows no selectivity. The latter control experiment shows that the enantioselective effect is due to the spin-exchange interaction, not due to the applied magnetic field. (**C**) ^1^H nuclear magnetic resonance (NMR) spectrum (400 MHz; H_2_O/D_2_O, 90:10) before and after the direct crystallization of RAO. Before the crystallization, the solution is a mixture of four aminoxazolines: RAO/AAO/LAO (f for furanose and p for pyranose)/XAO (42:30:18:10). After the crystallization, the redissolved crystals only contain RAO within the limits of ^1^H NMR detection. Thereby, mere crystallization on the magnetic surface stereoselectively and enantioselectively purifies RAO. ppm, parts per million.

**Fig. 3.**
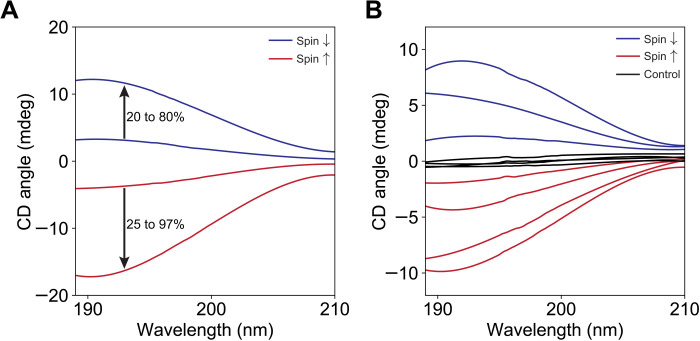
Recrystallization of enantioenriched and racemic RAO on magnetite. (**A**) When we crystallized enantioenriched solutions of RAO, we observed a marked increase in the enantiomeric excess (ee), and, from about 25%, ee we could reach a homochirality state. The 25% enriched D-RAO (red) is crystallized on an up-spin magnetite, and we obtained homochiral D-RAO. Similarly, 20% enriched L-RAO (blue) is crystallized on a down-spin magnetite, and we obtained nearly homochiral (80%) L-RAO. (**B**) Repeated recrystallization of racemic RAO is done to accumulate statistics. DL-RAO is crystallized on up- and down-spin magnetite. Red curves correspond to up-spin and blue curves to down-spin. Black lines are the control experiments done on a silicon surface in the presence of the magnetic field. The ee for the down-spin experiments is calculated to be 59, 36, and 18 (mean, 38%). The ee for the up-spin experiments is calculated to be −10, −22, −41, and −54 (mean, −32%). The ee for the control experiments is calculated to be 5.5, 0.9, 0.6, and −3.4 (mean, 0.9%). We estimate an error of ±5% for the ee calculations.

In this work, we demonstrate that the spin-polarized surfaces themselves can function as chiral agents, breaking the chiral symmetry in a further expansion of the role of the CISS effect (*30*–*32*). The simultaneous conglomerate crystallization provides the amplification of the induced ee at the surface, necessary to reach a homochiral state (Fig. 1). Here, we report the spin-selective homochiral crystallization of a ribonucleotide precursor, RAO, on magnetite surfaces, from its completely racemic solution (Figs. 2 and 3).

### Homochirality of RNA and the central role of RAO

RNA is thought to have played two major roles in the origin of life. The sequence of the nucleobases attached to the sugar phosphate backbone constitutes genetic information that can be passed from generation to generation by replication via a complementary strand through Watson-Crick base pairing. The sequence of an RNA also dictates its shape, and it is the adoption of a wide variety of shapes that endows RNA with its catalytic ability. Enantiomeric purity of the nucleotide components of RNA is crucial to both its roles. Replication via a complementary strand proceeds by way of an A-form duplex, and this is not possible if the nucleotide components deviate significantly from enantiomeric purity. Incorporation of a nucleotide of opposite handedness into an RNA strand changes its shape, and this might endow a different or improved catalytic ability, but the chirality switch cannot be passed to subsequent generations by replication, so the potentially beneficial change is nonhereditable. Establishing a mechanism whereby the nucleotide building blocks of RNA might have been synthesized enantiomerically pure is thus crucial to understanding how Earth’s RNA-based life originated. Peptides too depend on the enantiomeric purity of their component amino acids. In extant biology, the correlation of d-ribonucleotides with l-amino acids is established in two principal ways. Ribonucleotides and chiral amino acids are biosynthesized with high stereoselectivity, and l-amino acids are attached to transfer RNA (tRNA) composed of d-ribonucleotides by stereoselective aminoacyl-tRNA synthetases. The idiosyncratic behavior of amino acids makes the synthesis of all l-amino acids under early Earth conditions a daunting challenge, but recent findings suggest that this hurdle might be side-stepped. Prebiotically plausible chemical means of attaching amino acids to tRNA analogs have been found and proceed with high-level control of relative stereochemistry. Thus, for example, the l- over d-stereoselectivity for attachment of alanine to a tRNA acceptor stem mimic composed of d-ribonucleotides is of the order of 10:1 (*33*). This suggests that the overall homochirality problem of prebiotic chemistry might be reduced to the problem of making ribonucleotides in enantiopure form; racemic amino acids might suffice.

RAO emerged as a potentially important ribonucleotide precursor more than 50 years ago, but there remained several issues to be dealt with before its true potential was realized. Sanchez and Orgel (*20*) showed that RAO (which they synthesized from ribose and cyanamide) was a highly crystalline compound that underwent reaction with cyanoacetylene to generate α-cytidine, but subsequent conversion to the natural β-anomer was very inefficient. Furthermore, the synthesis of ribose is one of the persistent challenges of prebiotic chemistry; although the formose reaction is now very well understood, it still cannot be controlled to produce more than a trace of ribose as part of a complex mixture. Nevertheless, the attractiveness of RAO prompted further research, and Springsteen and Joyce (*21*) showed that it could be sequestered from simpler mixtures of sugars than those made by the formose reaction by reaction with cyanamide followed by crystallization. Joyce also reported more on the crystallization behavior of RAO and showed that it crystallized in the chiral *P*2_1_2_1_2_1_ space group and that crystals grown from solutions of *rac*-RAO were composed of twinned clusters with individually homochiral domains. The problem with the provenance of ribose was solved when Sutherland and colleagues showed RAO could be obtained, along with lesser amounts of the other pentose aminooxazolines, by reaction of glyceraldehyde with 2-aminooxazole, the latter itself deriving from the reaction of glycolaldehyde with cyanamide (*22*). The same group also found that nonracemic solutions of RAO gave rise to crystals with increased ees, and, above a threshold ee, enantiopure crystals were obtained. This behavior is consistent with a conglomerate susceptible to twinning. The conglomerate nature of RAO was lastly proven by Powner (*34*). Then, high-yielding prebiotic syntheses of ribonucleotides from aminooxazolines were reported by Sutherland and colleagues. First, it was shown that arabino-aminooxazoline could be elaborated to pyrimidine nucleoside cyclic phosphates (*23*); then, it was shown that RAO could be converted to pyrimidine nucleotides by a reaction sequence involving a photochemical inversion of the anomeric stereocenter (*25*). In the meantime, Blackmond and colleagues had shown that enantiomerically enriched proline could participate in the reaction of 2-AO with glyceraldehyde and give enantiopure RAO after crystallization (*17*). What has remained elusive, however, is how racemic compounds could give rise to enantiopure RAO purely by means of a process controlled only by the environment.

### CISS and spin-selective chemistry

The CISS effect has established a robust coupling of electron spin to molecular chirality. The initial experiments have shown that the electron transfer through a chiral monolayer is spin-dependent and that the preferentially transferred spin state depends on the handedness of the monolayer (*35*, *36*). The experiments have achieved near-perfect spin filtering at room temperature, showing the robustness of the coupling (*37*). Later work has proved that the coupling established by the CISS effect can be manifested in various ways from spintronic applications to long-range electron transfer in biology (*38*–*40*). More recent studies have shown that spin-selective behavior exists for freely diffusing molecules near magnetic surfaces (*41*, *42*) and even for achiral reagents (*43*, *44*). These studies have motivated us to consider the spin-selective processes near magnetic surfaces due to the CISS effect to impose a chiral bias on prebiotic chemistry.

Recently, we proposed closed-basin evaporative lakes with authigenic ferrimagnetic sediments (e.g., magnetite and greigite) as plausible prebiotic environments in which enantiospecific processes can be carried out because of the CISS effect (*28*). Our mechanism used UV-ejected photoelectrons from magnetite surfaces as chiral agents in reductive synthesis because of the helical character of such electrons in the close vicinity of the magnetic surface [figure 1 of (*28*)]. We proposed that these helical electrons can achieve kinetic resolution in the reduction of chiral molecules for which the reaction rates for enantiomers differ by $\exp\left(\frac{2H_{\mathrm{SO}}}{k_{B}T}\right)$. Here, *H*_SO_ is defined as the effective coupling due to a combination of spin-orbit and spin-exchange interactions of a chiral molecule with an electron, *k*_B_ is the Boltzmann constant, and *T* is the temperature [figure 2 of (*28*)]. As a suitable chemistry to apply our idea, we proposed cyanosulfidic chemistry that uses photoejected hydrated electrons to drive the reductive synthesis of sugars (*24*, *45*). In particular, we focused our attention on the spin-selective reduction of the cyanohydrin of glycolaldehyde that is producing glyceraldehyde, the first chiral sugar, and an RNA precursor, after the hydrolysis of the resultant imine. Although this proposed reduction is still a worthy experiment, the isomerization of glyceraldehyde to dihydroxyacetone and the cyanide liberating equilibrium of the reagent cyanohydrin make it a harder reaction to study. Moreover, freely diffusing molecules with a single chiral center are likely to display less enantioselective behavior due to the reduced coupling of the electron spin to the molecular frame. Therefore, in this work, we studied the crystallization of the stable RNA precursor, RAO, as a static process with higher selectivity, on a magnetic surface.

**Fig. 4.**
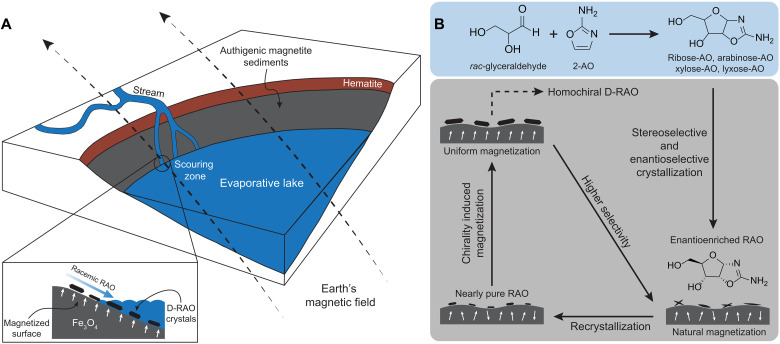
An evaporative lake with magnetic sediments can accommodate spin-selective processes between an RNA precursor and a magnetized surface. (**A**) An evaporative lake contains authigenic magnetite sediments magnetized by the Earth’s magnetic field. An incoming stream with racemic aminooxazolines scours the muddy material off the surface. As the lake evaporates, D-RAO selectively crystallizes on the magnetic surface. (**B**) An incoming stream carries the reaction products of racemic glyceraldehyde and 2-aminooxazole: racemic RAO, AAO, XAO, and LAO. Initially, the magnetic sediments are authigenically magnetized and carry a bias along the Earth’s field. This natural bias enables the enantioselective crystallization of RAO, which also sterically purifies RAO from the mixture of aminooxazolines. After a small enantiomeric imbalance is induced, sporadic water flow redissolves the RAO crystals, and, in a subsequent dry phase, RAO recrystallizes on the surface with higher enantiomeric excess (ee). As these nearly pure conglomerate crystals accumulate more material, they cover a larger area of the surface and flip the spin of magnetic domains along the chiral molecular axis. This chirality-induced magnetization process increases the magnetization of the surface and allows for a positive feedback loop between the magnetic surface and the chiral crystals. As a result, the surface with higher magnetization induces higher ee for the upcoming crystallizations, and, eventually, enantiopure D-RAO is obtained.

We should emphasize that, if realized, enantioselective synthesis of glyceraldehyde reinforces the spin-selective crystallization of RAO as glyceraldehyde’s chirality directly determines the chirality of RAO as can be seen in the blue box in Fig. 4B. However, the crystallization process does not rely on starting from an enantioenriched solution of RAO; therefore, our results presented here are self-sufficient to achieve a homochiral RNA world.

### Magnetic surfaces as chiral agents

Magnetic surfaces themselves can be chiral agents due to the CISS effect, and surface processes such as crystallization or adsorption on a magnetic surface can be enantioselective. As demonstrated by the early CISS experiments, electron flow from a surface through a chiral monolayer is spin-dependent. The same effect manifests itself when a chiral molecule acquires an induced charge polarization because the latter is nothing but a transient electron flow inside a chiral potential. In addition, because of the CISS effect, charge polarization is accompanied by spin polarization. This transient, induced charge polarization can be due to intermolecular interactions among chiral molecules or between a chiral molecule and a surface (*39*, *46*).

When a chiral molecule approaches a surface, the electron density of the molecule redistributes itself, which gives rise to an induced charge dipole. In addition, for a chiral molecule, this transient electron flow is spin-dependent due to the CISS effect and gives rise to a transient spin polarization as shown in Fig. 1A. This spin polarization is realized along the chiral molecular axis, and it is dependent on the handedness of the chiral molecule. In Fig. 1A, the right-handed (D) enantiomer has the minority spin (↓) on its positively charged pole (δ^+^), whereas the left-handed (L) one has the majority spin (↑). This is how the chiral symmetry is broken by a magnetic surface, which is itself spin-polarized: The spin-exchange interaction between the surface electron spins and the transiently spin-polarized chiral molecules is higher or lower depending on the handedness of the molecule. The favorable, lower energy interaction with the surface corresponds to a singlet-like (↑↓), and the penalized, higher energy interaction corresponds to a triplet-like (↑↑) overlap of the spins. Therefore, a spin-polarized surface kinetically traps an enantiomer based on its spin polarization and breaks the chiral symmetry. In other words, a magnetized surface can be considered as a crystal seed, breaking the chiral symmetry by promoting the crystallization of one enantiomer. However, it should be noted that this is a kinetic entrainment–like phenomenon and not a thermodynamic effect. Therefore, if all molecules are allowed to crystallize, then the enantioselective effect cannot be observed.

Enantioselective behavior on magnetic surfaces due to the CISS effect has been demonstrated with the adsorption of chiral molecules like double-stranded DNA and l-cysteine on magnetized ferromagnetic films (*30*). Moreover, enantioseparation on magnetic surfaces by conglomerate crystallization has been realized by Tassinari *et al.* (*31*) with a moderate ee. This pioneering study lays the groundwork for our current research, as we use the same effect with a higher efficiency while also ensuring its relevance to prebiotic conditions. Therefore, we use magnetite (Fe_3_O_4_) surfaces available on early Earth and stable under ambient conditions and work with a compound (i.e., RAO) central to the synthesis of RNA, from which the propagation of homochirality to the entire prebiotic network is possible.

Naturally, the CISS-driven interaction between chiral molecules and magnetic surfaces work both ways around: A chiral molecule can also selectively spin polarize a magnetic surface and the direction of this polarization is determined by the handedness of the chiral molecule. This effect has also been demonstrated by the chirally selective magnetization switching in a ferromagnet upon the adsorption of chiral molecules (*47*). In that work, Ben Dor *et al.* (*47*) have shown that chiral molecules can robustly affect the magnetization of surfaces due to the long-range spin-exchange interaction. Chirality-induced magnetization switching combined with the presented results pave the way for positive feedback between chiral molecules and magnetic surfaces that can purify the magnetization of the iron-oxide sediments and thus promote the homochiral crystallization of RAO.

### Feedback between the magnetic surface and RAO

Using the CISS-based interaction between magnetic surfaces and chiral molecules, it is possible to envision a positive feedback loop between the magnetite surfaces and RAO in a prebiotic setting as shown in Fig. 4B.

Magnetite is the most abundant natural magnetic mineral on Earth, as well as the strongest one (*48*, *49*). In a wide range of natural depositional environments, authigenic magnetite sediments form and magnetize under the Earth’s field, resulting in a chemical remanent magnetization (*50*). Sediments that acquired a chemical remanence after the most recent geomagnetic reversal are expected to have a statistically uniform remnant magnetization on a hemisphere scale within a shallow depth interval (*51*). This initial natural bias of the magnetic domains can break the chiral symmetry if racemic RAO crystallizes on the magnetic surface. However, because of the imperfect alignment of the magnetic domains, the first crystallization attempt can only induce a small enantiomeric imbalance. However, after the enriched crystals are dissolved due to a sporadic water flow and recrystallize on the magnetic surface again, the selectivity will be higher due to the asymmetric crystallization of RAO (*22*) and further magnetic seeding due to the CISS effect (Fig. 4). When these nearly pure RAO conglomerates grow on the surface, they can simultaneously interact with the surface spins and switch the magnetization of the surface. This process can purify the magnetic domains along the chiral molecular axis of RAO. In addition, because these sedimentary rocks are no longer small superparamagnetic particles, this CISS-induced magnetic diagenesis can permanently lock the surface spins along one direction, unless a large (much larger than Earth’s field) coercive field is applied. When the surface spins are locked once and for all, they can induce efficient enantioselectivity for the upcoming crystallizations and the magnetized surface can separate homochiral RAO crystals in just a few cycles.

This feedback mechanism can be more effectively realized over a small area such as the scouring zone of an incoming stream as shown in Fig. 4A. At the scouring zone, the authigenic magnetic sediments are exposed, wet-dry cycles are more frequently realized (like a shore), and an incoming stream can constantly feed the surface with racemic RAO. With the described feedback mechanism, magnetized magnetite sediments at the scouring zone can filter out homochiral RAO crystals from the incoming racemic stream.

## RESULTS

We have studied the crystallization of RAO from its racemic solution on magnetite surfaces. We fabricated the magnetite surfaces as thin films (200 nm) on silicon substrates following the procedure by Jubb and Allen (*52*) (see also section S5). We used electron beam evaporation to deposit 100-nm iron on silicon (100) surface and then heated the sample at 175°C for 4 hours to promote the oxidation of iron, producing a magnetite film of about 200 nm. We characterized the samples by Fourier transform infrared spectroscopy and confirmed the complete conversion of iron to magnetite (fig. S5). We further analyzed the surface roughness and magnetic properties of the samples by atomic force microscopy (AFM) and superconducting quantum interference device (section S6). We placed the magnetite surfaces horizontally in a petri dish and placed a magnet just below the surface as shown in Fig. 1B. We used the magnet to spin-polarize the magnetite surface and measured the magnetic field to be 325 mT at the surface location. It is important to place the magnet such that the magnetization direction is parallel to the surface normal to maximize the helical character of the surface electrons.

The ^1^H nuclear magnetic resonance (NMR) spectra confirmed the stereoselective crystallization of RAO, as has been previously reported (*21*, *22*). Having identified the composition of the crystals by NMR, we measured the chiro-optical properties by circular dichroism (CD) spectroscopy. We obtained a nonzero CD signal for the individual needle-shaped crystals of RAO; however, the rosette-shaped crystals did not consistently give a CD signal. We further analyzed the rosette-shaped crystals by x-ray crystallography and found that the individual arms of the rosettes contain only one enantiomer yet the crystal as a whole is racemic. Therefore, we found that the rosettes formed because of the twinning of needle-shaped homochiral domains. When we analyzed rosettes with less homochiral domains, we obtained a nonzero CD signal with a randomly varying sign. However, the rosettes with more branching did not give a CD signal. We also did a control experiment by crystallizing the enantiopure compound and still observed the formation of rosette-shaped crystals. Therefore, we concluded that the twinned, rosette-shaped crystals formed because of a stochastic arrangement of homochiral domains. Because this stochastic twinning occurs on an existing crystal face, it nullifies the enantioselection seeded by the magnetic surface. For the experiments in which we primarily obtained needles on the surface (fig. S12), we observed a surface-wide ee (in addition to the ee of the individual crystals); however, for most of the cases, we could not consistently avoid the formation of the twinned crystals. Therefore, we did not observe consistent selectivity for direct crystallization experiments on magnetite surfaces. However, if the rosette formation can be avoided by the change of conditions, such as concentration, buffer, or pH, then it should be possible to observe selectivity for direct crystallization.

We proceeded with the recrystallization of RAO. We synthesized racemic RAO by the reaction of d- and l-ribose with cyanamide on a large scale. We confirmed that synthesized RAO is fully racemic with a CD measurement before the crystallization experiments (fig. S18). We then prepared a 65 mM solution of the racemic RAO in pure water and placed it on the magnetized magnetite surface. We followed the same crystallization procedure described above and obtained diamond-shaped crystals on the magnetite surface as shown in Fig. 2A. We collected the crystals with tweezers, dissolved them in pure water, and obtained their CD spectra: first individually and after for the whole surface. We confirmed that diamond-shaped crystals of RAO are individually homochiral by CD and x-ray diffraction measurements. These diamond-shaped crystals and the individual needle-shaped ones both gave identical x-ray diffraction patterns, and they all belong to the *P*2_1_2_1_2_1_ chiral space group. However, with the diamond-shaped crystals, we did not observe twinning when the crystallization is stopped soon after the crystals are observed. When we waited longer, as the crystals grew in size, we observed that a crystal face became a seed for the other enantiomer. However, these twinned crystals mostly contained two to three macroscopic, homochiral domains unlike the rosettes containing many arms, for which each arm is an individual, homochiral domain. In addition, for the recrystallization experiments, twinned diamond crystals appeared well after the individual diamonds were formed and visible; therefore, we could stop the experiment before the twinning started to take over. However, for the direct crystallization experiments, rosettes appeared almost simultaneously with the needles so there was no slow progression of the twinning that we could control.

Having confirmed that diamond-shaped crystals are individually homochiral, we dissolved all the crystals on the magnetite surface together in water and obtained the CD spectrum for the whole surface. To our delight, not only the individual crystals showed optical activity but also the entire surface did. As the smoking gun of the CISS-driven phenomenon, when we reversed the magnetic field direction (therefore, the spin state of the surface electrons), the observed CD signal reversed in sign. As shown in Fig. 2B, left-handed RAO crystals (blue curve) dominated the down-spin (↓, south pole) surface and the right-handed ones dominated the up-spin (↑, north pole) surface. We obtained an ee of about 60% for the entire surface, for both spin directions starting from a completely racemic solution of RAO. (Check section S8.3 on how the ee is calculated.) For this step, it was crucial to collect the crystals early, as the enantioselectivity of the magnetic surface is a kinetic entrainment effect, and, beyond a certain point, ee decreased with the increasing crystallization yields and due to enantiomorphous twinning. Crystals were usually visible after several hours, and we collected them after about 6 hours to a day. We collected 48 and 16 individual crystals for the up-spin and down-spin experiments, respectively, for the data shown in Fig. 2B. Because of the high number of crystals and the repeatability of the results (Fig. 3B), we can rule out the presence of a net ee due to statistical fluctuations of homochiral crystals.

We investigated the reason why recrystallization of RAO gives crystals with different morphology (diamond-shaped) compared to direct crystallization of the reaction products of glyceraldehyde and 2-aminooxazole (needle-shaped) and crystallized RAO with added aminooxazolines [arabino-AO (AAO), lyxo-AO (LAO), and xylo-AO (XAO)] one by one. We found that, in the presence of XAO, RAO crystallizes as needle-shaped crystals, whereas AAO and LAO do not visibly modify the crystallization habit of RAO. Moreover, we observed a concentration-dependent formation of the rosette-shaped twinned crystals only when RAO is crystallized in the presence of XAO (fig. S26). At higher concentrations, when XAO is present in the solution, RAO forms rosette-shaped crystals, and the crystal morphology is altered by the relative amount of XAO (fig. S26); therefore, we conclude that XAO is a crystal habit modifier for RAO [see scheme 1 of (*53*)]. We also found that, at higher relative concentrations (e.g., 1:1), XAO is embedded in the crystal lattice structure of RAO as an impurity with 4.8(4)% abundance, as detected by x-ray diffraction analysis (fig. S31). Last, we found that XAO affected the RAO crystallization only when the relative stereochemistries matched (e.g., D-RAO with D-XAO); we did not observe any effect of XAO when we crystallized L-RAO with D-XAO. These findings show the necessity of recrystallization to observe enantioenrichment to enantiopurity, as, by removing XAO from the solution, racemizing stochastic twinning is circumvented.

As a control experiment, we recrystallized RAO on a nonmagnetic, silicon surface in the presence of a magnetic field of the same strength, and we collected the crystals from the nonmagnetic surface and obtained the black spectra in Fig. 2B. As seen, the surface with no net spin polarization does not induce any enantioselectivity in the presence of a magnetic field, as shown by Pasteur (*1*). The control experiment confirms that the observed selectivity is not due to the magnetic field but due to the spin-exchange interaction. The control experiments on the nonmagnetic surface and the consistent flipping of the ee (Fig. 3B) with the flipping magnetic pole direction ensure that the obtained ee is physical and not due to the contamination of surfaces with chiral impurities.

We repeated the recrystallization experiment on magnetite for both pole directions multiple times and found that, on average, we can get around 35% ee for the entire magnetite surface. We then considered this value as a typical outcome from a racemic solution and then crystallized the enriched crystals on the magnetic surface one more time. We found that, above a starting ee of about 25%, we can obtain completely enantiopure crystals (Fig. 3A). Therefore, in just two crystallization steps on the magnetic surface, we could achieve homochirality from a completely racemic mixture.

## DISCUSSION

We have demonstrated an efficient mechanism to resolve racemic RAO, an important RNA precursor, on a prebiotically available mineral surface, magnetite (Fe_3_O_4_), and obtained homochiral crystals of RAO in two crystallization steps.

Our mechanism features the two requisites to reach homochirality: chiral symmetry breaking by the magnetic surface due to the CISS effect and self-amplification by conglomerate crystallization. The symmetry breaking by magnetic surfaces is prebiotically plausible, nondestructive, and robust at room temperature and in solution. Although the demonstrated symmetry breaking is a surface effect [two-dimensional (2D)], conglomerate crystallization allows for the extension into the bulk (3D). Therefore, the attained selectivity at the surface level can be seamlessly carried into the bulk of the solution.

Our mechanism requires a well-defined magnetic surface (fig. S10), and the crystallization on colloidal magnetic particles is not enantioselective. A detailed discussion on this can be found in section S7.1. In light of this fact, we consider sedimentary rock surfaces similar to those found in the Gale crater (*54*), as opposed to colloidal muds, as likely sites to realize our mechanism. In these environments, magnetite is present as inclusions in silica-based rocks (magnetite-silica facies) and is highly magnetic, especially as single-domain particles (*49*, *54*). In such a setting, we envision a kinetically favored crystallization of RAO on magnetized magnetite inclusions.

With the feedback effect that we suggested, our mechanism offers a persistent and deterministic chiral bias to prebiotic chemistry as opposed to a singular and stochastic trigger (e.g., spontaneous symmetry breaking). The advantage of this over a spontaneous one is that, at various stages of prebiotic chemistry, the same chiral bias is present in the environment in a persistent basis, reinforcing the previously attained ee. In addition, by combining the symmetry breaking with a well-matched, simultaneous amplification, we have shown a direct way to reach the homochiral state from a completely racemic starting point.

The mechanism is a kinetic entrainment–like effect, and the magnetic surface acts as a chiral seed based on its magnetization direction. Because of the kinetic nature of the effect beyond a certain point, enantioselectivity will decrease with increasing crystallization yields, as the system approaches to the thermodynamic equilibrium. Because of this, the mechanism can benefit from the presence of flow as previously considered by Ritson *et al.* (*55*). As such, we can conceive of the enantioselective crystallization of RAO on the scouring zone of an evaporative lake by an incoming stream carrying the racemic solution of sugar aminooxazolines. In this scenario, the crystallization takes place on the shallow shores of the magnetite lake, which can undergo multiple wet-dry cycles, allowing several crystallization-dissolution cycles of RAO. The flow also scours the soft, muddy material off the surface and exposes the authigenic magnetite sediments on which enantioselective processes can take place. Moreover, while the flow feeds the magnetic surface with a racemic solution of RAO, it simultaneously washes the other enantiomer in the solution and the racemic powder suspended in the bulk away from the zone and allows for higher enantioselectivity.

It seems more convenient to conceive of the feedback loop that we propose on a smaller region rather than the whole surface of the lakebed. With selective crystallization and magnetization cycles, the spin purity of the magnetic surface around the scouring zone can be locked with less material, and this small bottleneck region over which the material flows can act as a chiral filter for the incoming RAO. Nevertheless, achieving our enantioseparation mechanism is not a fine-tuned geochemical scenario, and one can come up with other (or more refined) prebiotically plausible scenarios compatible with this process. We, therefore, emphasize the robustness and the strength of our mechanism rather than the specific geochemical scenario that can accommodate the enantioseperation process.

The efficiency of the enantioseparation by magnetic surfaces is concentration-dependent, and it works best around or below the solubility limit of the compound. At higher concentrations, the selectivity goes down as the eutectic equilibrium forces the crystallization. Therefore, the slower the crystallization process and the lower the concentration, the higher the enantioselectivity. The low solubility of RAO in water allowed for obtaining its crystals at low concentrations; therefore, we could achieve high enantioselectivity in just one crystallization in comparison to the previous work by Tassinari *et al.* (*31*) with highly soluble amino acids in water. Our results can be further improved if the magnetic surface is placed vertically such that the crystals forming in the bulk do not fall on the magnetic surface and reduce the selectivity, as demonstrated by Bhowmick *et al.* (*32*).

In addition, we used magnetite as the magnetic substrate with near-unity spin polarization at its Fermi level (*56*) in comparison to commonly used Ni/Au substrates with lower intrinsic spin polarization (around 20%) and protective gold layer further reducing the spin polarization (*31*, *32*). Therefore, we have shown that magnetite surfaces can be used effectively in the CISS experiments due to their optimal spin polarization properties.

Similar to the previous work by Tassinari *et al.* (*31*), we used magnetic substrates to resolve chiral compounds, yet our work is constrained by prebiotic relevance, and our results differ from theirs in multiple aspects. First, we obtain higher ee’s and achieve homochirality due to the low solubility of RAO in water and high spin polarization of magnetite. Because of the high solubility of conglomerate-forming amino acids in water, Tassinari *et al.* (*31*) used supersaturated solutions prepared at 80°C, resulting in lower ee’s. In contrast to this, RAO is poorly soluble (∼0.01 g/ml) in water, and its conglomerate crystallization does not require supersaturation or extreme conditions that are prebiotically implausible. In addition, they achieve the selective crystallization of racemic amino acids from their aqueous solutions only by asparagine as they could not resolve racemic threonine, and glutamic acid is resolved as its hydrochloric acid (HCl) salt in high concentrations of HCl. This harsh condition is prebiotically unlikely, and HCl is known to dissolve magnetite, creating iron chlorides. Therefore, the pioneering work by Tassinari *et al.* (*31*) establishes the feasibility of the enantioseperation mechanism using magnetic substrates however does not account for the origin of biological homochirality.

Although we used stronger magnetic fields to spin-polarize the magnetite surfaces than what was likely available on early Earth, similar spin polarizations under weak fields can be achieved if magnetite forms as small (∼100 nm in diameter) superparamagnetic particles. Such authigenic magnetite particles have been shown to have remanent magnetizations of above 10 kA/m even when they form under weak magnetic fields (0.1 mT ≈0.08 kA/m) comparable to the Earth’s (*49*). Because our mechanism is based on spin-exchange interaction rather than a magnetic field effect, the figure of merit is not the magnetic field strength but the degree of spin alignment (magnetization) at the active surface. Hence, one should pay attention to the prebiotically relevant remanent magnetizations available in natural magnetic minerals rather than early Earth’s magnetic field strength. Having said that, with the described feedback mechanism between the chiral molecules and the magnetic surface, even higher magnetizations can be realized.

RAO is not just like any chiral compound; it has a central role in the synthesis of ribonucleotides, and its chirality directly determines the chirality of RNA. The attained homochirality at RNA can then be spread to peptides by the stereoselective binding of amino acids to the tRNA analogs (*33*). Moreover, RAO is very stable against isomerization unlike sugars like ribose or glyceraldehyde as well as against thermal degradation and UV damage. Therefore, it is a very suitable molecule to lock the chirality in prebiotic chemistry. Combining this with the fact that it crystallizes as a conglomerate, RAO is an ideal molecule to apply the described enantioseparation mechanism. However, as shown by Tassinari *et al.* (*31*), the mechanism is versatile and applicable to other chiral molecules forming conglomerate crystals (e.g., asparagine).

## MATERIALS AND METHODS

Reagents and solvents were obtained from Acros Organics, Santa Cruz Biotechnology, and Sigma-Aldrich and were used without further purification unless otherwise specified. We performed all of the experiments under ambient conditions unless otherwise specified.

### Method for synthesizing RAO, AAO, XAO, and LAO

Ribo-, arabino-, xylo-, and lyxo-aminooxazolines were synthesized by the reaction of two equivalents of cyanamide with one equivalent of the corresponding aldopentose sugar. Cyanamide (5 g, 0.12 mol, 2 eq) was added to a solution of sugar (9 g, 0.06 mol, 1 eq) in aqueous ammonia (3.5%, 10 ml). Then, the resultant mixture was swirled at room temperature, and all solid material was dissolved. After 30 min, the solution was maintained at 60°C for another hour. The reaction mixture was then cooled to room temperature, and methanol (10 ml) was added to promote crystallization. After 16 hours at 4°C, the crystals were collected by filtration, washed with ice-cold methanol (20 ml), and dried under vacuum.

We separately purchased the L and D sugars and synthesized the L and D aminooxazolines in large amounts (gram level for each enantiomer). We then weighed the enantiomer on the scale, ground them into powder, and mixed them in equal amounts to make the racemic pentose aminooxazolines. We confirmed that the aminooxazolines are racemic by CD measurements.

### Method for fabricating magnetite surfaces

We fabricated the magnetite films by evaporating a 100-nm iron layer on 0.625-mm-thick silicon (100) wafers using electron beam evaporation under a high vacuum of 5 × 10^−6^ torr. Following the evaporation, we baked the samples at 175°C for 4 hours in the air and promoted the oxidation of iron (Fe) to magnetite (Fe_3_O_4_). We then cleaned the sample surfaces with acetone and subsequently in ethanol before every experiment.

### Method for crystallization experiments

For the direct crystallization experiments, we prepared a 0.5:0.5 M solution of racemic glyceraldehyde and 2-aminooxazole in 2 ml of water. We incubated the solution at 40°C for 12 hours and obtained a yellow-brown solution of aminooxazoles. For the recrystallization experiments, we prepared a 65 mM solution of racemic RAO in 2 ml of water.

We placed the magnetite surfaces horizontally in a polystyrene petri dish (35 mm by 10 mm) on a magnet such that the surface normal is parallel to the magnetization direction. The magnetic field strength at the sample position was measured to be 325 mT with a Hall probe. We then filled the petri dish with the incubated solution and made sure that the magnetite surface is covered with the liquid. We then placed the setup in the fridge kept at 12°C and waited until the first crystals appear. This process can take several hours to a few days. Afterward, we slowly filtered out the mother liquid and washed three times the surface and crystals with pure water such that the racemic liquid is washed away. We then collected the crystals with tweezers under a stereomicroscope. We discarded the crystals formed on the plastic surface of the petri dish and on the rough edges of the silicon substrate and collected the rest formed on the magnetite surface. We fully dissolved all of the collected crystals in pure water and analyzed the solution.

### Method for CD measurements

We took the CD measurements in a quartz cuvette after diluting the solution in 2 ml of water until the UV/visible (Vis) absorption peak is below optical density of 1 for accurate measurements. We used the Jasco J-815 Circular Dichroism Spectropolarimeter with an active temperature control connected to a water bath with a temperature set to 20°C. The temperature feedback is performed by a Jasco PFD-425S/15 controller with a Peltier control unit. Before the measurements, we took a baseline measurement of the water and cuvette background. We simultaneously measured the CD and UV/Vis absorption of the sample together with the photomultiplier voltage to ensure that the spectrometer is not operating beyond its specified voltage range of 600 units. We took the measurements in the 185- to 210-nm wavelength range and used the auto-baseline subtraction feature. We used a data pitch of 0.2 nm, a bandwidth of 1 nm, a data integration time of 1 s, and a scanning speed of 20 nm/min. We averaged each measurement five times. We normalized the CD signal amplitude on the basis of the UV/Vis absorption and calculated the ee using the calibration procedure described in section S9 (*57*–*62*).

## Supplementary Materials

### This PDF file includes:

Materials and Methods Sections S1 to S11 Figs. S1 to S32 Tables S1 to S11 Legend for movie S1 Legends for data S1 to S3
