# Supplementary material for: Origin of biological homochirality by crystallization of an RNA precursor on a magnetic surface
Source: Sci Adv. 2023 Jun 7;9(23):eadg8274. doi: 10.1126/sciadv.adg8274 (PMC10246896; doi:10.1126/sciadv.adg8274)
Supplement: Supplementary file 1 — Materials and Methods Sections S1 to S11 Figs. S1 to S32 Tables S1 to S11 Legend for movie S1 Legends for data S1 to S3 [file sciadv.adg8274_sm.pdf]

Science Advances
AAAS

Supplementary Materials for

Origin of Biological Homochirality by Crystallization of an RNA Precursor on a Magnetic Surface

S. Furkan Ozturk *et al.*

Corresponding author: S. Furkan Ozturk, sukrufurkanozturk@g.harvard.edu

Sci. Adv. **9**, eadg8274 (2023)
DOI: 10.1126/sciadv.adg8274

The PDF file includes:

Materials and Methods
Supplementary Text
Figs. S1 to S32
Tables S1 to S11
Legend for movie S1
Legends for data S1 to S3

Other Supplementary Material for this manuscript includes the following:

Movie S1
Data S1 to S3

1. Materials and general methods

Reagents and solvents were obtained from *Acros Organics*, *Santa Cruz Biotechnology*, and *Sigma-Aldrich*, and were used without further purification. A *Mettler Toledo* SevenEasy pH Meter S20 combined with a *ThermoFisher Scientific* Orion 8103BN Ross semi-micro pH electrode was used to measure and adjust the pH to the desired value. ^{1}H-Nuclear magnetic resonance (NMR) spectra were acquired using a *Bruker* Ultrashield 400 Plus or *Bruker* Ascend 400 operating at 400.13 MHz. The notations s, d, t, and m represent the multiplicities singlet, doublet, triplet, and multiplet signal, respectively. Chemical shifts (δ) are shown in ppm.

2. Syntheses of RAO, AAO, and XAO

Cyanamide (5 g, 0.12 mol, 2 eq) was added to a solution of sugar (9 g, 0.06 mol, 1 eq) in aqueous ammonia (3.5 %, 10 mL). The resultant mixture was swirled at room temperature until all solid material was dissolved. After 30 min, the solution was maintained at 60ºC for a further 1 hour. The reaction mixture was then cooled to room temperature and methanol (10 mL) was added to promote crystallization. After 16 hours at 4ºC the crystals were collected by filtration, washed with ice-cold methanol (20 mL) and dried under vacuum.

D-RAO (yield: 85 %): ^{1}H-NMR (400 MHz, Deuterium Oxide) δ 5.73 (d, *J* = 5.1 Hz, 1H), 4.91 (t, *J* = 5.3 Hz, 1H), 4.05 (dd, *J* = 9.6, 5.5 Hz, 1H), 3.86 (dd, *J* = 12.7, 2.4 Hz, 1H), 3.66 (dd, *J* = 12.7, 4.8 Hz, 1H), 3.54 (ddd, *J* = 9.6, 4.7, 2.4 Hz, 1H).

L-RAO (yield: 84 %): ^{1}H-NMR (400 MHz, Deuterium Oxide) δ 5.72 (d, *J* = 5.0 Hz, 1H), 4.90 (t, *J* = 5.3 Hz, 1H), 4.05 (dd, *J* = 9.5, 5.5 Hz, 1H), 3.85 (dd, *J* = 12.7, 2.4 Hz, 1H), 3.65 (dd, *J* = 12.7, 4.8 Hz, 1H), 3.54 (ddd, *J* = 9.5, 4.7, 2.4 Hz, 1H).

D-AAO (yield: 40 %): ^{1}H-NMR (400 MHz, Deuterium Oxide) δ 5.82 (d, *J* = 5.5 Hz, 1H), 4.84 (d, *J* = 5.5 Hz, 1H), 4.24 (d, *J* = 3.3 Hz, 1H), 3.93 (td, *J* = 6.0, 3.4 Hz, 1H), 3.52 (dd, *J* = 12.1, 5.4 Hz, 1H), 3.45 (dd, *J* = 12.2, 6.9 Hz, 1H).

L-AAO (yield: 21 %): ^{1}H-NMR (400 MHz, Deuterium Oxide) δ 5.83 (d, *J* = 5.6 Hz, 1H), 4.85 (dd, *J* = 5.6, 1.5 Hz, 1H), 4.25 (dd, *J* = 3.5, 1.6 Hz, 1H), 3.94 (ddd, *J* = 6.9, 5.4, 3.4 Hz, 1H), 3.53 (dd, *J* = 12.2, 5.4 Hz, 1H), 3.46 (dd, *J* = 12.2, 6.9 Hz, 1H).

D-XAO (yield: 59 %): ^{1}H-NMR (400 MHz, Deuterium Oxide) δ 5.86 (d, *J* = 5.2 Hz, 1H), 4.81 (d, *J* = 5.1 Hz, 1H), 4.29 (d, *J* = 2.4 Hz, 1H), 3.86 – 3.68 (m, 3H).

L-XAO (yield: 58 %): ^{1}H-NMR (400 MHz, Deuterium Oxide) δ 5.86 (d, *J* = 5.1 Hz, 1H), 4.81 (d, *J* = 5.1 Hz, 1H), 4.29 (d, *J* = 2.4 Hz, 1H), 3.86 – 3.67 (m, 3H).

3. Syntheses of LAO

Lyxose (855 mg, 5.7 mmol) was dissolved in H_2O and cyanamide (492 mg, 11.7 mmol) was added. The pH of the solution was adjusted to 6.7 with NaOH (1 M) and it was heated at 60ºC for 5 hours before lyophilization. The resultant oil was purified by flash column chromatography (eluting with methanol/ethyl acetate 0:1 to 1:4). Those fractions containing aminooxazoline were combined and concentrated under vacuum.

D-LAO(p): ^{1}H-NMR (400 MHz, Deuterium Oxide) δ 5.48 (d, *J* = 5.5 Hz, 1H), 4.88 (dd, *J* = 5.5, 3.4 Hz, 1H), 3.51 (dd, *J* = 13.2, 4.1 Hz, 1H). The rest of the signals for D-LAO(p) overlap with those of D-LAO(f) between δ 3.78 - 4.00.

D-LAO(f): ^{1}H-NMR (400 MHz, Deuterium Oxide) δ 5.69 (d, *J* = 5.8 Hz, 1H), 5.12 (dd, *J* = 5.8, 3.4 Hz, 1H), 3.58 (dd, *J* = 13.4, 3.4 Hz, 1H). The rest of the signals for D-LAO(f) overlap with those of L-LAO(p) between δ 3.78 - 4.00.

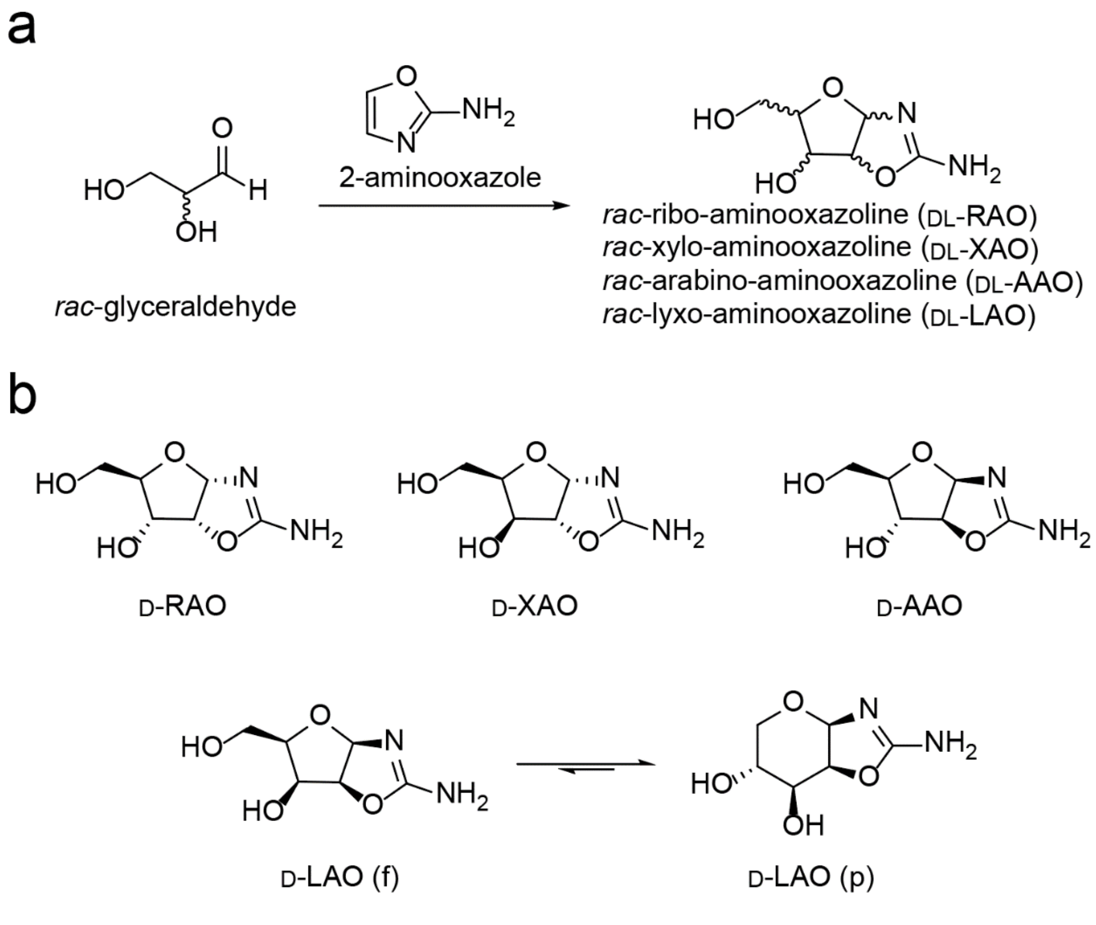

Fig. S1. a. Synthesis of the mixture of racemic aminooxazolines involves incubation of the racemic sugar, DL-glyceraldehyde, with 2-aminooxazole. The racemic mixture of aminooxazolines is left to crystallize for the direct crystallization experiments. **b.** Chemical structures of the right-handed ribo-, xylo-, arabino-, and lyxo-aminooxazolines. Lyxo-aminooxazoline has two structural isomers in equilibrium: the five-membered ring furanose and the six-membered ring pyranose.

4. Syntheses of aminooxazolines from glyceraldehyde for direct crystallization

DL-glyceraldehyde (90.08 mg, 1 mol) was dissolved in 2 mL H_2O and 2-aminooxazole (84.08 mg, 1 mol) was added. The resultant mixture was then stirred until a homogenous solution was obtained. This solution was then incubated at 40ºC for 24h and during which time it developed a yellow-brown coloration. The pH of the incubated mixture was measured to be 8.5. The incubated mixture was then analyzed by ^1H NMR spectrometry before the crystallization experiments and the relative ratios of aminooxazolines were determined by integration.

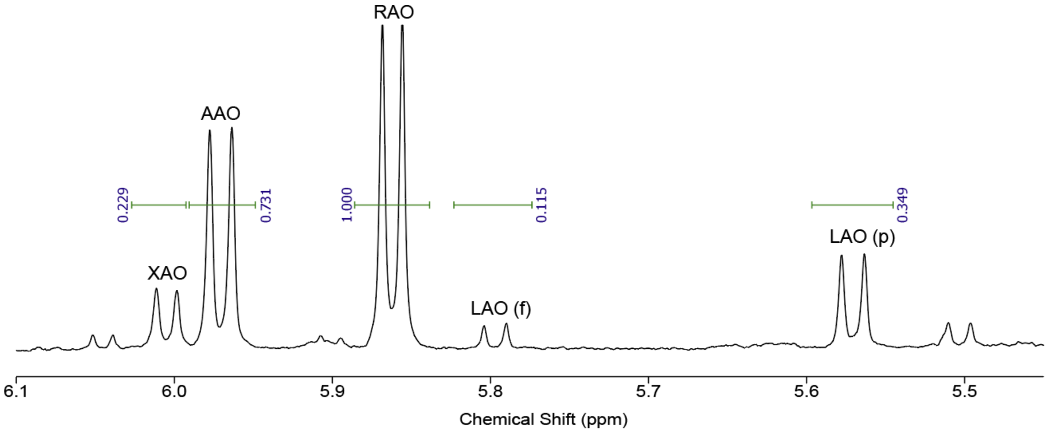

Fig. S2. ^1H NMR (400 MHz, Deuterium Oxide) spectra of racemic aminooxazolines synthesized by the incubation of *rac*-glyceraldehyde and 2-amiooxazole. Integrated area under the doublet peaks is shown.

5. Fabrication of magnetite (Fe_3O_4) surfaces

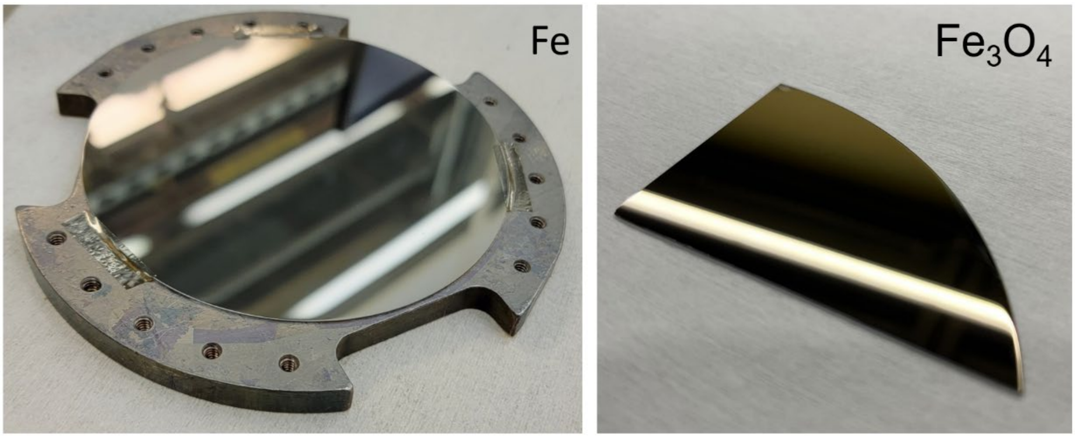

Fig. S3. 100 nm thick iron (Fe) evaporated on a silicon substrate (100 mm diameter, 500 µm thickness) gives a metallic, shiny gray surface (left). After the oxidation of the film in air at 175ºC for 4 hours, iron oxidizes to a smooth film of about 200 nm magnetite, Fe_3O_4, with a green-yellow-black color (right).

We fabricated the magnetite films using the procedure demonstrated by Jubb and Allen (2010) (*52*). We first deposited iron films by the electron-beam evaporation of iron on a silicon substrate. We used a 150 mm diameter wide and 625 µm thick boron doped P type Si <100> wafers from University Wafers (catalog ID number 3784). For the evaporation, we used SHARON EE-3 e-beam evaporation system located at the Harvard Center for Nanoscale Systems (CNS). A

cryopump and an oil-free roughing pump provide the evaporator vacuum. We used a FABMATE® crucible liner made out of high-strength graphite by Kurt J. Lesker (Part number: EVCFABEB-13) to place the iron as an e-beam source. As the iron source, we used iron 99.95% pure iron pellets (1/8" diameter by 1/8" long) by Kurt J. Lesker (Part number: EVMFE35EXED). We filled the crucible liner with the iron pellets and placed it inside the e-beam evaporator, together with the silicon substrate as the target. We vacuumed the system down to 5 x 10^{-6} Torr before we started the deposition process. We used the density of 7.86 g/cm³ and the z ratio of 0.349 for the iron evaporation. We turned on the e-beam source after the vacuum is stabilized and waited until a stable deposition rate of 0.5 Å/s is reached. We then opened the target shutter and started the iron deposition. We used a quartz crystal to monitor the *in-situ* thickness of the iron film and slowly deposited 100 nm of iron. We found that the slower the deposition rate the smoother the resultant iron film. We found an optimal deposition rate of around 0.2-0.7 Å/s. We then took the iron sample on the silicon substrate out of vacuum and placed it inside an oven (Binder Model FD 56 Laboratory Oven) set at 175ºC under ambient air. We oxidized the iron film for 4 hours to magnetite (Fe_3O_4) and observed that the shiny gray iron film turned into a smooth layer of greenish-yellow, black magnetite film, as seen in Fig. S3. According to the cited procedure (*52*), 100 nm of iron should give around 200 nm of Fe_3O_4 after the oxidation due to oxygen incorporation into the iron lattice.

After the magnetite films were fabricated, as a precautionary measure, we kept them in a vacuum desiccator to prevent the further oxidation to hematite and hydrous iron oxides. However, the films were stable under oxic, ambient conditions for weeks and we have not observed any physical damage or chemical degradation while doing experiments in water solutions.

6. Characterization of magnetite (Fe_3O_4) surfaces

6.1 AFM

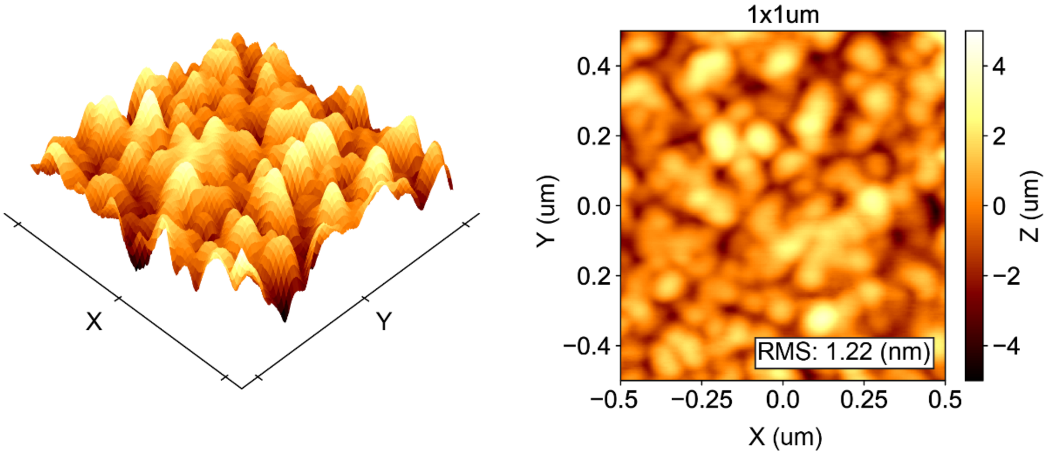

Fig. S4: AFM topography image (3D image on the left, 2D top view on the right) of a magnetite film prepared from 100 nm iron deposited on a silicon wafer, with a slow deposition rate of 0.5 Å/s. Peak-to-peak surface roughness of 8.93 nm and an RMS roughness of 1.22 nm is measured for a randomly picked 1 x 1 µm area.

We characterized the surface properties of the magnetite films with a commercial AFM (Cypher S by Asylum Research) located at Harvard CNS facility. We used the AC mode (dynamic mode) and we imaged in air. We used a cantilever (Nanosensors Type SSS-NCH-10) with a nominal force constant of 42 N/m and a resonance frequency about 330 kHz. The cantilever oscillated at the auto-tuned resonance close to its frequency. The scan parameters were set at a scan rate of 1.00 Hz, an integral gain of 30.00, and a setpoint of 800.00 mV. The tip's amplitude and phase were monitored with a photodetector by using the tip deflection. We took scan ranges of 1.0 x 1.0 µm, 5.0 x 5.0 µm, and 10.0 x 10.0 µm and calculated the root-mean-square (RMS) roughness from the 3D topography data.

Scan Range (µm)	Peak-to-peak (nm)	RMS Roughness (nm)
1.0 x 1.0	8.93	1.22
5.0 x 5.0	11.42	1.41
10.0 x 10.0	10.82	1.30

Table S1: Peak-to-peak variations and RMS surface roughness are shown for three different scan ranges, randomly picked on a magnetite surface.

6.2 FTIR

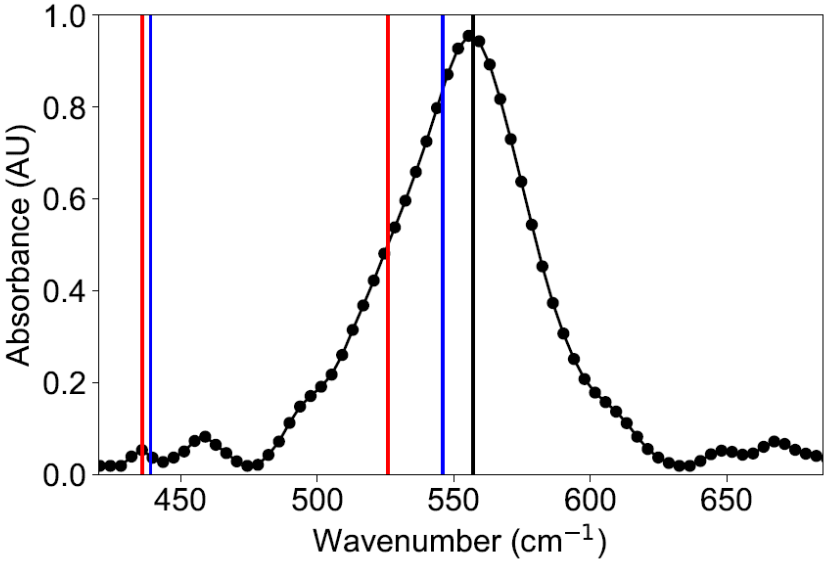

Fig. S5: FTIR spectra of the 40 nm Fe_3O_4 films on 5 mm thick KBr substrate. The prominent peak centered at 557 cm^{-1} (black line) is due to the magnetite absorption. Vertical blue and red lines are the expected locations of the maghemite and hematite respectively. FTIR spectra shows that iron films are selectively oxidized into magnetite.

We characterized the vibration al spectra of magnetite films by Fourier transform infrared (FTIR) spectroscopy. For FTIR measurements, we used a potassium bromide (KBr) window by ThorLabs (25 mm diameter, 5 mm thick, Part number: WG10255). We chose this material due to its low absorption in the mid-IR range. We coated the KBr window with a 20 nm iron film using e-beam evaporation as described in Supplementary Information Section 5. When we oxidized the sample, we obtained a 40 nm magnetite film according to Jubb and Allen (2010) (*52*). We used a commercial FTIR spectrometer by Bruker (Model: Invenio, 8000 cm^{-1}

to 350 cm^{-1}). For the measurements, we used a resolution of 5 cm^{-1}, sample scan average of 100 scans, phase resolution of 16 in the Mertz phase correction mode. We used a MIR source and a KBr beam-splitter with a RT-DLaTGS detector. We took the measurements in the range of 700-350 cm^{-1}. We placed a KBr window into the background arm and the KBr/40 nm Fe_3O_4 sample into the sample arm of the spectrometer and used the background subtraction feature of the spectrometer. The measurement gave a prominent peak centered at 557 cm^{-1} (black vertical line in Fig. S5) with a full width at half maximum of around 50 cm^{-1}, as shown in Fig. S5. The central location of the peak agrees very well with the phonon mode splitting of magnetite, Fe_3O_4, as observed by Jubb and Allen (560 cm^{-1}) and theoretically calculated by Glotch et al. (558 cm^{-1}) [57]. We have not observed spectroscopic features of maghemite (439 and 546 cm^{-1}, blue vertical lines in Fig. S4) and hematite (436 and 526 cm^{-1}, red vertical lines in Fig. S4). We were not able to observe the magnetite mode at 350 cm^{-1} due to high background KBr absorption and we attribute the small ripples in the background to the residual water IR absorption. The absorption intensity we measure is not physical due to the reflection losses from the shiny magnetite film. FTIR spectra shows that iron films are selectively oxidized into magnetite and not into other iron oxides like maghemite (much less magnetic than magnetite) and hematite (not magnetic).

6.3 UV-VIS

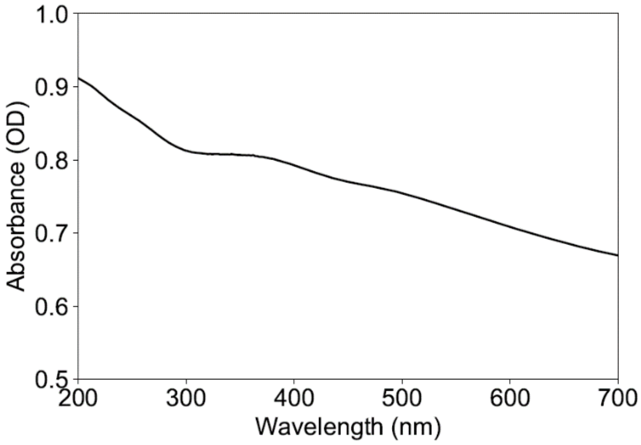

Fig. S6: UV-Vis spectra of the 40 nm Fe_3O_4 films on 1 mm thick fused silica substrate. Magnetite absorbs prominently in the visible range and it has a broad absorption feature slowly increasing into deep UV.

We measured the optical spectra of magnetite films by ultraviolet–visible (UV-Vis) spectroscopy. For UV-Vis measurements, we used an uncoated, broadband UV fused silica window by ThorLabs (25.4 mm diameter, 1 mm thick, Part number: WG41050). We chose this material due to its low absorption in the optical range. We coated the fused silica window with a 20 nm iron film and when we oxidized the sample we obtained a 40 nm magnetite film according to Jubb and Allen (2010) (*52*). We used a commercial UV-Vis spectrometer by Shimadzu (Model: UV-1900, 190 nm to 1100 nm). We took the measurements in the 190 – 700 nm range with a resolution of 2 nm, using the slow acquisition mode. We placed a fused

silica window into the background arm and the fused silica/40 nm Fe_3O_4 sample into the sample arm of the spectrometer and obtained the background subtracted UV-Vis spectra. The measurement gave a broad absorption feature, slowly rising as the wavelength decreased as seen in Fig. S6.

6.4 SQUID

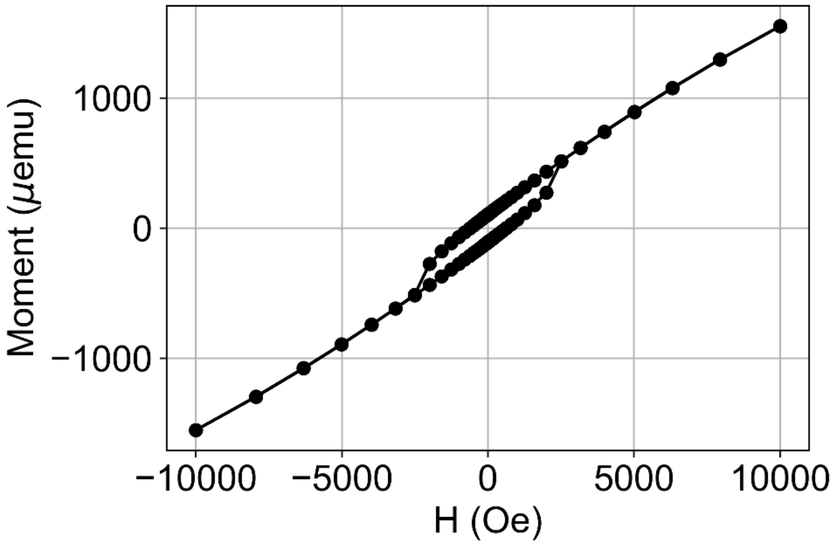

Fig. S7: Hysteresis curve of a 200 nm magnetite sample on a 625 μm thick (4 mm by 4mm) Si wafer. The coercive field is measured to be around 600 Oe and as seen the sample does not saturate at 10 kOe field.

We measured the magnetic properties of the magnetite samples by a superconducting quantum interference device (SQUID). We used the 200-nm-thick magnetite samples for the SQUID measurements on the silicon substrate. We took the measurements at room temperature by magnetizing the sample parallel to the surface normal. We separately measured the silicon substrate and subtracted its diamagnetic contribution. We measured the hysteresis curve of the sample with a magnetizing run from 0 Oe to 10 kOe, a first measurement from 10 kOe to -10 kOe, and a second one back to 10 kOe, ending with a demagnetizing run from 10 kOe to 0 Oe. We used a scan rate of 50 Oe/s. We measured the coercive field to be around 600 Oe, confirming the soft magnetic nature of magnetite. In addition, we observed that the sample does not saturate even at fields as high as 10 kOe, reaching a moment of above 1000 μemu. Around the fields the crystallization experiment were conducted (~ 3000 Oe) the moment was measured to be around 600 μemu.

7. General method for crystallization experiments

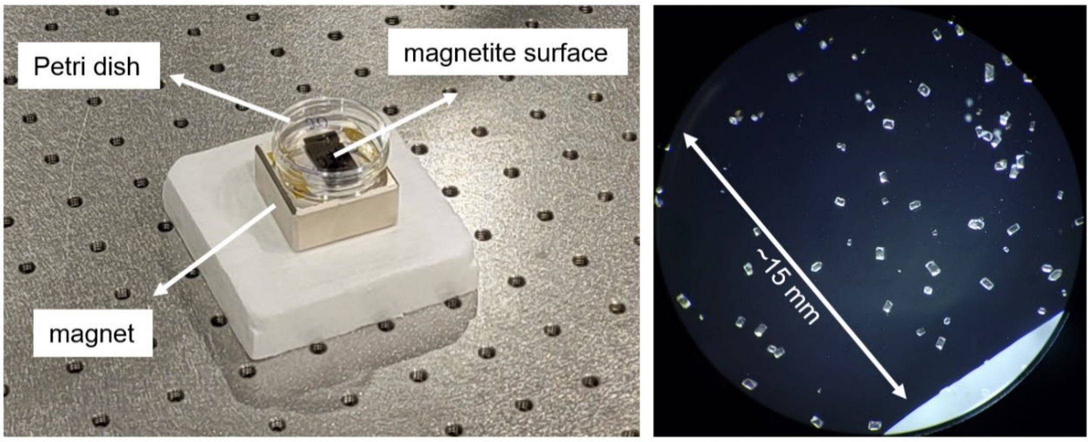

Fig. S8: Photo on the left shows a typical experimental setup. Photo on the right shows the magnetite surface after a typical re-crystallization experiment with diamond-shaped crystals formed on the magnetic surface.

For the crystallization experiments we cut the magnetite wafers such that they could fit inside a 35 mm diameter Petri dish (Falcon Corning, Polystyrene, SN:351008, VWR#:25373-041) used for the experiments. We typically used samples with dimensions around 20 mm height by 15 mm width. Before every experiment we wiped the magnetite samples with acetone and then with ethanol to remove any organic residues and contaminants. We then secured the magnetite samples in a Petri dish horizontally by a double-sided kapton tape such that they are stable. Before we added the solution, we blew the samples with clean air to get rid of any dust particles, which can act as nucleation sites for crystallization and reduce enantioselectivity. We then placed the Petri dishes containing the magnetite samples on top of a strong magnet (DIYMAG 40 x 40 x 20 mm N52 Neodymium magnet) such that the magnetization direction of the magnet is parallel to the magnetite surface normal. Before placing the surface on the magnet we marked the pole of the magnet so that we knew the spin direction for the particular experiment. North pole corresponds to surfaces polarized parallel to the surface normal and the South pole corresponds to surface polarized anti-parallel to the surface normal. We measured the magnetic field strength and the pole direction (North or South) with a Hall probe (Extech MF100 AC/DC magnetic field meter). For the reported experiments, we have used a magnetic field strength of 325 mT at the magnetite sample location as measured by the magnetic field meter. Afterwards, we placed the Petri dish with the magnet underneath into a fridge kept at 12ºC. After placing the magnets, we waited for about 10 minutes so the samples reached thermal equilibrium. Then we prepared a 2 mL racemic solution of RAO by fully dissolving the powdered material in warm water and slowly put the solution on the magnetite surface making sure that whole surface is covered with the solution, and no air gap is left. We have not filtered the solution before placing on the surfaces with a filter (e.g., 0.22 um pore size filter membrane), in order to decrease the number of possible nucleation sites due to the fact that a non-filtered solution is more representative of the natural

prebiotic conditions. However, in principle, we think that using filtration paper or membrane can enhance the number of enantioselective crystallizations seeded on the magnetite surface. After we placed the solutions, we closed the Petri dish with its lid and did not disturb the experiment for about 24 hours, in order not to contaminate it. Typically, after 24 hours to 48 hours we stopped the crystallization and slowly collected the solution above the surfaces together with the floating racemic powder inside the water column. We then washed the surfaces with cold water several times to get rid of the racemic liquid and the floating crystals. We used cold water such that we could clean the surface without dissolving the crystals attached to the surface. After the surface has been cleaned, we waited for about 30 minutes such that the crystals dry and were easier to collect. We then collected the crystals individually under a stereomicroscope (Amscope 7x-45x, SM-2T-6WB-V331) using tweezers. We recorded how many crystals we collected for each surface.

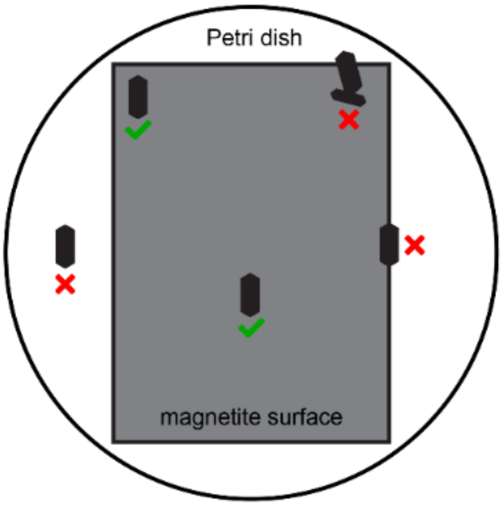

Fig. S9: After a typical experiment, the crystals forming on the active area of the magnetic surface are collected. Crystals forming outside of the magnetic surface or directly at the rough edges of the surface, or the ones seeded by the crystals formed on the rough edges of the surface are not collected.

We should mention that we collected the crystals formed on the magnetic surface and we discarded the ones forming on the plastic areas of the Petri dish and the crystals formed on the rough edges of the silicon wafer as shown in the schematic in Fig. S9. After collecting the crystals, we fully dissolved them in pure water for CD experiments. We occasionally discarded experiments if they had a contamination acting as a nucleation site such as visible pieces of lint or dust. We observed that if large pieces of dust are floating inside the solution they aggregate many crystals on top of each other. These aggregated crystals drop on the magnetic surface due to gravity and invalidate the experiment. Another crucial point is to find an optimal time to stop the crystallization. Because the enantioselective crystallization effect is a kinetic one, after a point increasing crystallization yields decreases selectivity. Furthermore, because the solution is not kept always racemic, if one enantiomer is depleted too much from the solution with selective

crystallization the relative concentration of the other increases and this promotes the crystallization of the opposite enantiomer.

Finally, yet importantly, once a crystal forms and grows, its face can act as a seed for the opposite enantiomer forming twinned racemic clusters nullifying the selectivity seeded by the surface. We found that the slower the crystallization and the earlier the crystals are collected the higher is the enantioselectivity. Optimal time may vary from experiment to experiment, but as a general guideline, collecting the crystals as soon as they are visible by eye and collectible by tweezers maximizes the measured enantioselectivity. Finally, if the experiments are started in a “fresh” room with fewer air seeds available, the optimal conditions may vary after a while when the room becomes saturated with the air seeds of the aminooxazoline. Although air seeding is an anecdotal phenomenon, we observed that the initial experiments required slightly higher (about 10-15% higher) concentrations for enantioselective crystallization compared to the final, optimal conditions. We attribute this to the fact that we started the experiments in a fresh laboratory in which RAO molecule had not previously been studied; therefore, no crystal seeds of RAO were available in the air.

7.1 Importance of using smooth magnetic surfaces

Before we started using the smooth thin films of magnetite made by e-beam evaporation, we experimented with magnetite powder and spin-coated magnetite nanoparticles and we have not observed the enantioselective effect. In the case of magnetite powder precipitated on the bottom of a cuvette due to gravity, the particles on the surface are still freely rotating. And therefore both sides of each particle surface are exposed although the magnetic field direction is pointing only one way. This is like the case described in the left panel of Fig. S10. When a

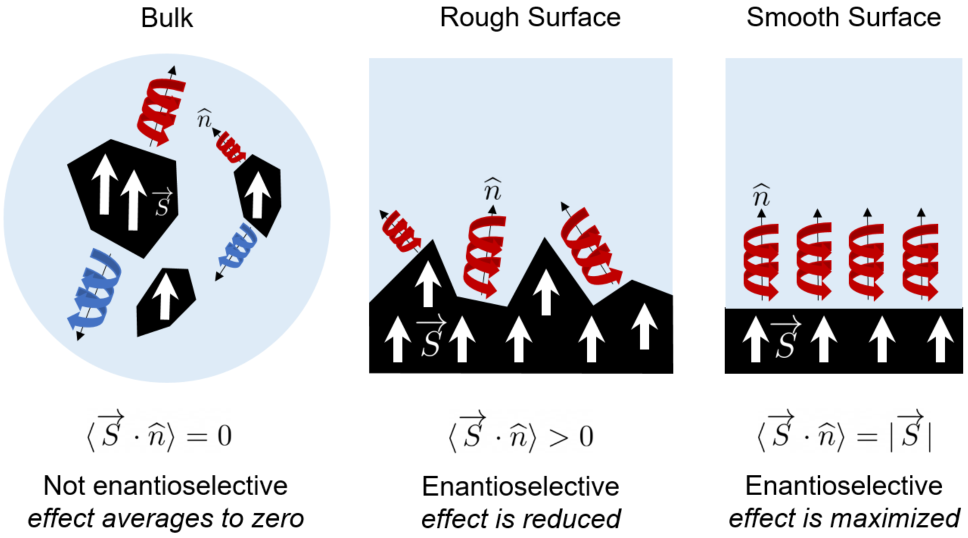

Fig. S10: In the bulk, a freely diffusing magnetic particle cannot induce selective crystallization due to isotropy. A well-defined magnetic surface breaks chiral symmetry by allowing for seeding only on the upper half plane. For a rougher surface, net selectivity exists but the strength of the effect is reduced, due to the averaging of spin-surface normal overlap.

chiral molecule approached a magnetic surface parallel or antiparallel to the direction of the magnetic field, no net selective effect is observed as on the upper face one enantiomer is selectively crystallized and on the lower face, the opposite enantiomer is crystallized. Therefore, a loose, soft sediment is like a freely diffusing particle in the bulk in terms of being a seed for selective crystallization. For the case of a well-defined but a rough surface (middle column of Fig. S10) the situation is different. For rough surfaces with net spin alignment, depending on the spin vector's overlap with the "averaged" surface normal there is a net selective effect. However, for a rough surface, the surface normal is not uniform and one has to average the spin overlap for all locally flat surfaces. Therefore, the enantioselective effect is reduced although the chiral symmetry is still broken by the magnetic surface. For an ideal surface with perfect uniformity enantioselective effect is maximized as the spin vectors can perfectly overlap with the surface normal therefore with the molecular frame of chiral molecules. Of course, in a natural scenario, there maybe no such thing as a perfectly smooth surface. Therefore, the selectivity achieved with pristine surfaces should be large, as we have

demonstrated, such that in a natural scenario the effect persists on rougher surfaces albeit with diminished magnitude.

7.2 Direct crystallization of DL-RAO from the mixture of aminooxazolines

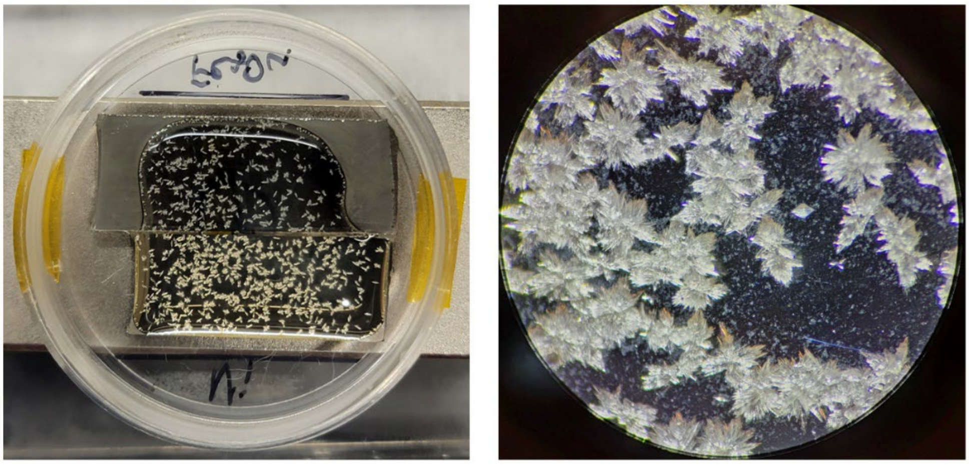

Fig. S11: A direct crystallization experiment (left) and the zoomed in photo of the magnetite surface (right) after it is covered by the rosette-shaped crystals.

We first synthesized a racemic mixture of aminooxazolines by incubating 0.5 M DL-glyceraldehyde with 0.5 M 2-aminooxazole in 2 mL pure water at 40ºC for 24 hours. We then put the incubated solution on a magnetized magnetite surface and allowed for crystallization. Typically, within a few hours we started to observe needle-shaped crystals appearing on the

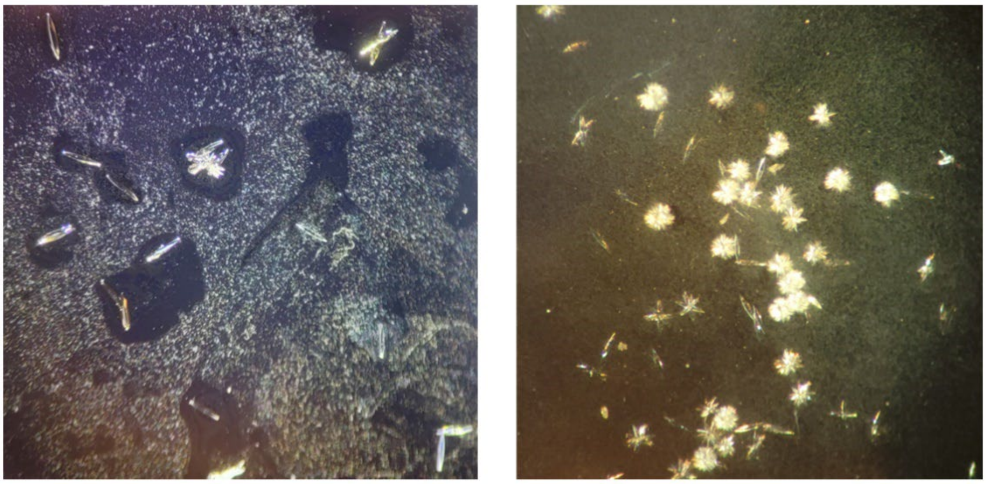

Fig. S12: Photos of the magnetite surface after two different direct crystallization experiment. On the left, a surface level ee is achieved due to the low number of twinned clusters. Individual needles are homochiral and they are seeded by the magnetic surface. On the right, no enantioselectivity is achieved due to the crystal twinning in the form of rosette shaped clusters. Note the powdery racemic material which can be removed along with the mother liquor by aspiration (mimicking the effect of drainage in a geochemical setting).

magnetite surface almost simultaneously with the rosette-shaped ones as seen in Fig. S12. We stopped the crystallization as soon as the crystals were visible however, we could not reliably control the formation of rosette-shaped twinned clusters. We then collected the crystals one-by-one and dissolved them in 2 mL pure water. We first confirmed that they

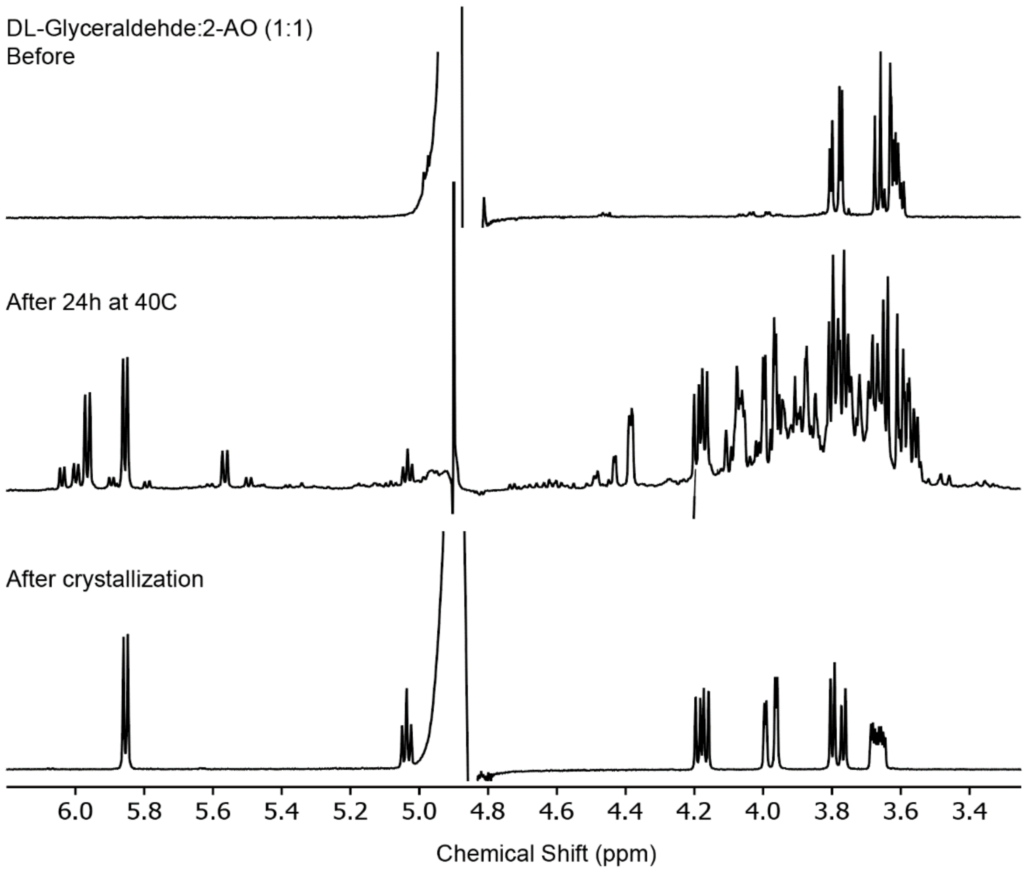

Fig. S13: ^{1}H NMR spectra of the starting solution (top row), after the incubation for 24 hours at 40 degrees (middle row), and after direct crystallization (bottom row).

consists of only RAO by ^{1}H NMR, within the limits of NMR detection. For NMR studies, we added 10% D_2O to our solutions.

We then checked the circular dichroism spectra of the solution. For direct crystallization, we could not reliably get a net CD signal. We then realized that the rosette-shaped crystal clusters forming from the racemic solution are racemic, twinned crystals of RAO with individual homochiral domains. Therefore, the twinning nullified the effect seeded by the surface, so no net enantiomeric excess was measured. We think that, if concentration is optimized and crystallization is slowed down, cluster formation can be circumvented and a net effect can be observed. For the control experiments, we crystallized the same starting solution on silicon substrates (without magnetite on them), in the presence of magnetic field. For this case, we did not observe large needles and rosettes but a flaky, white powder of racemic RAO covering the silicon surface, as shown in Fig. S14. Under a high-magnification

microscope, we have observed that the flaky powder actually consists of tiny needle-shaped crystals of RAO.

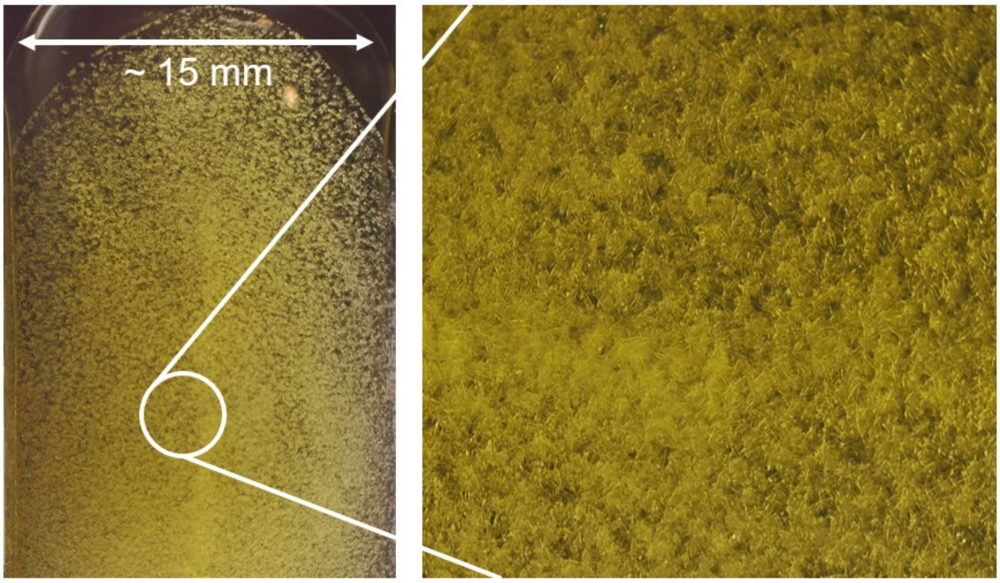

Fig. S14: When direct crystallization of the racemic mixture is done on a non-magnetic silicon surface, RAO crystallizes as a flaky powder and covers the surface. The zoomed in photo shows that the powder consists of tiny needle-shaped crystals.

7.3 Re-crystallization of DL-RAO

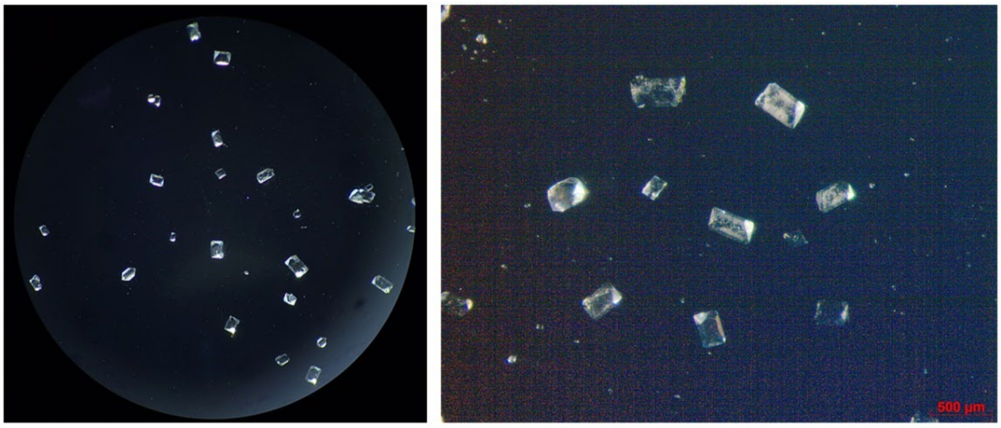

Fig. S15: After a typical re-crystallization experiment, we obtain diamond-shaped, shiny crystals of RAO on magnetite surfaces. These crystals, when collected early, are not twinned and they are seeded by the magnetic surface which thus shows a surface-wide ee.

We made a 65 mM solution of DL-RAO in 2 mL pure water. We then put the solution on magnetized magnetite and we typically started observing crystal formation in about 12 hours as shiny speckles on the surface. We collected the crystals one by one with tweezers usually in 24 to 48 hours depending on the existing crystal amount on the surface. We observed that the earlier we collected the crystals the higher the selectivity we obtained. There is typically a tradeoff between selectivity and the amount of crystals collected and we did not to collect less than 10 crystals for an experiment as due to small number statistics high ee's can be

deceptively obtained. After we collected the crystals, we fully dissolved them together in pure water and obtained their CD spectra. Table S2 shows the repeated experiments with different pole directions to accumulate statistics. The North pole (up spin) selects the crystals of D-RAO and the South pole selects the crystals of L-RAO. As one can notice, even for high number of individually homochiral crystals we collected (e.g., experiments # 1, 3, 7) we reliably observed spin-selective crystallization with high overall enantiomeric excess. CD spectra of the experiments displayed in Table S2 can be seen in Fig. 3 in the main manuscript and the raw data is shown on Fig. S16.

Experiment	Magnet Pole	Surface	%ee Before	%ee After	Crystal Number	Time It Took (h)
**1**	NORTH	Fe_3O_4	0	-54	48	24
**2**	NORTH	Fe_3O_4	0	-41	11	48
**3**	NORTH	Fe_3O_4	0	-22	82	36
**4**	NORTH	Fe_3O_4	0	-10	52	72
**5**	SOUTH	Fe_3O_4	0	59	16	24
**6**	SOUTH	Fe_3O_4	0	36	19	48
**7**	SOUTH	Fe_3O_4	0	18	47	36
**8**	NORTH	Si	0	5.5	9	48
**9**	NORTH	Si	0	0.6	16	72
**10**	SOUTH	Si	0	-3.4	12	48
**11**	SOUTH	Si	0	0.9	23	72
**12**	NORTH	Fe_3O_4	-25	-97	26	24
**13**	SOUTH	Fe_3O_4	20	80	18	24

Table S2: We repeated the re-crystallization experiments once the conditions were optimized and obtained consistent results confirming the spin-selective crystallization on magnetite surfaces. Red and blue rows of the table correspond to the experiments reported in the main manuscript.

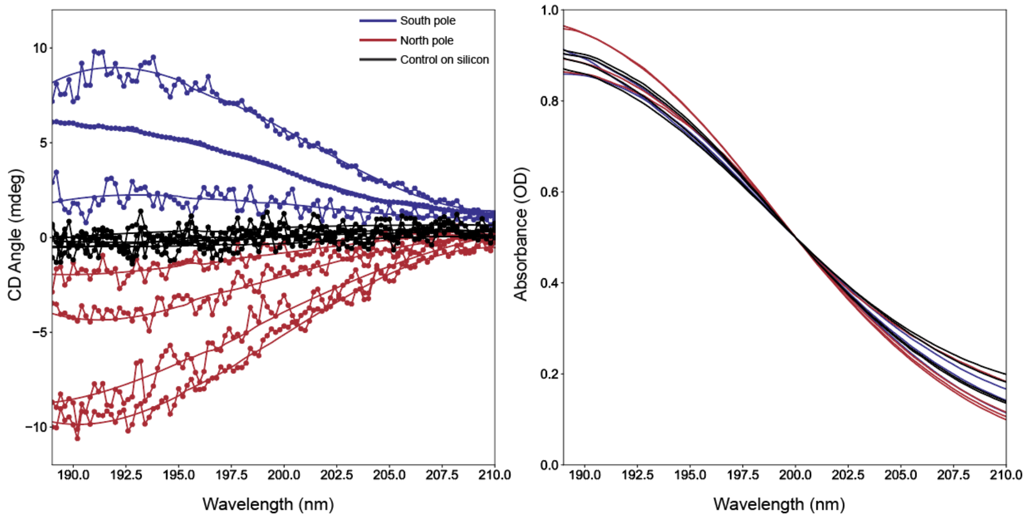

Fig. S16: Raw CD and UV-Vis spectra of the experiments shown in Table S2.

7.4 Re-crystallization of enantioenriched RAO

We prepared enantioenriched solutions of D- and L-RAO by mixing the racemic DL-RAO powder with the enantiopure D- and L-RAO powder. We measured the ee of the starting

solutions to be -25% and 20% for D-RAO and L-RAO respectively. We then crystallized the solution enriched with D-RAO on the North pole (up spin polarized magnetite) and the solution enriched with L-RAO on the South pole (down spin polarized magnetite). After re-crystallization, we found that the solution with -25% ee gave 26 crystals of enantiopure D-RAO. And for the solution with 20% ee we obtained an increased ee of 80% after the second crystallization. The raw data for the crystallization of the enantioenriched solutions can be seen in Fig. S17.

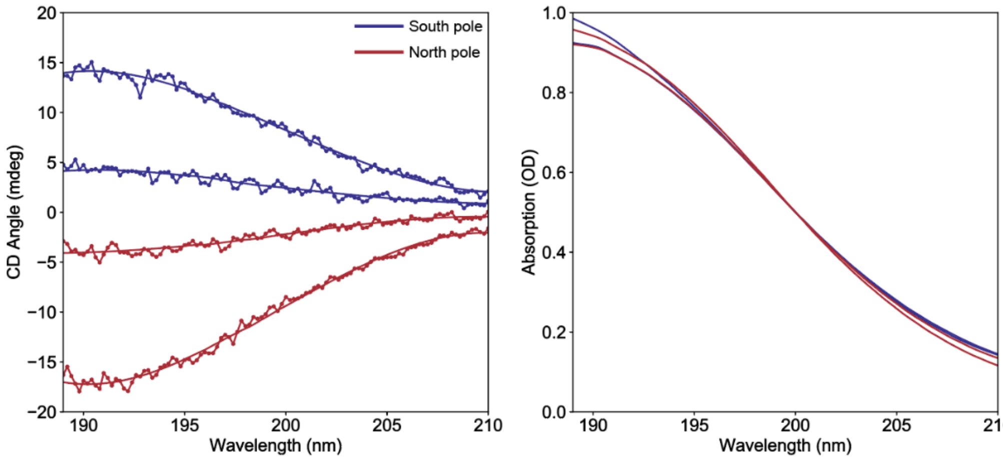

Fig. S17: Raw CD and UV-Vis spectra of the re-crystallization experiments from the enantioenriched solutions of RAO. (Experiments #12 and #13 in Table S2)

8. Circular dichroism (CD) measurements

8.1 General method for CD measurements

After we collected the crystals, we fully dissolved them in 2 mL of pure water. We took the CD spectra of the dissolved solution in a quartz cuvette with a path length of 10 mm (Starna Cells Inc 1-Q-10-GL14-C). Before each measurement we took the CD and UV-Vis spectra of the water-cuvette background and subtracted this background from the sample measurements using the auto-baseline subtraction feature of the spectrometer. We also made sure that the absorption peak of the solutions is below OD = 1 for accurate measurements in the linear regime of the spectrometer. We took the measurements using a Jasco J-815 Circular Dichroism Spectropolarimeter with an active temperature control connected to a water bath with a temperature set to 20ºC. We performed the temperature feedback by a Jasco PFD-425S/15 controller with a Peltier control unit. We simultaneously measured the CD, and UV/Vis absorption spectra of the samples together with the photo-multiplier voltage to ensure that the spectrometer is not operating beyond its specified voltage range of 600 units. We took the measurements in the 185-210 nm wavelength range. We used a data pitch of 0.2 nm, a bandwidth of 1 nm, a data integration time of 1 second, and a scanning speed of 20 nm/minute. We averaged each measurement 5 times.

8.2 CD measurements of the starting racemic solutions

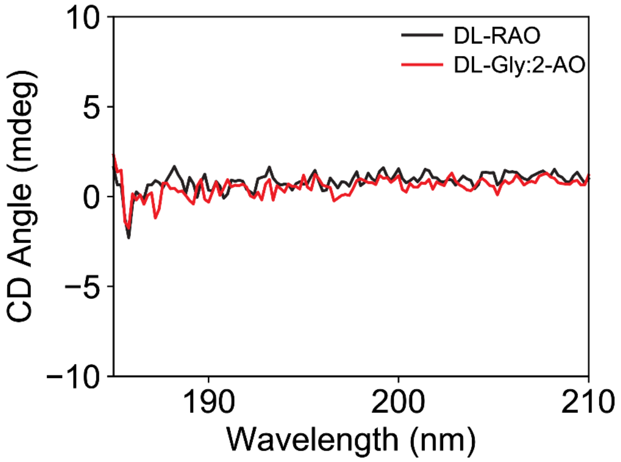

Fig. S18: CD spectra of the synthesized DL-RAO (black) and the incubated solution synthesized from DL-glyceraldehyde:2-AO (red). The spectra shows that the starting solutions for the direct crystallization and re-crystallization experiments are racemic.

We obtained the CD spectra of the synthesized DL-RAO and the incubated solution synthesized from DL-glyceraldehyde before the crystallization experiments to make sure that the starting solution is racemic, within the limits of the CD measurement.

8.3 Enantiomeric excess calibration curves

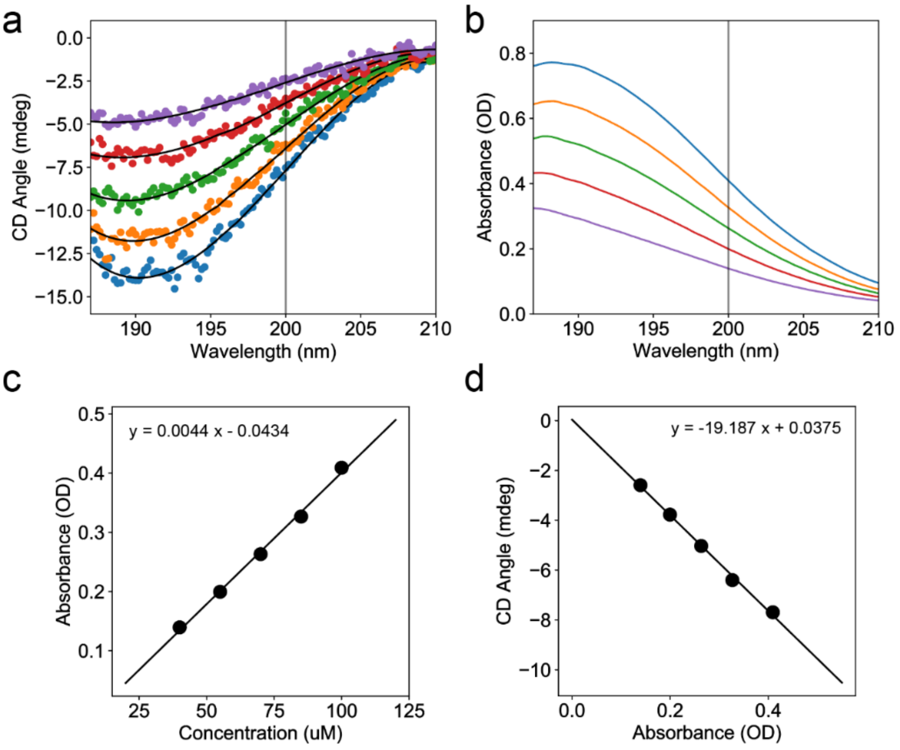

Fig. S19: a, b) CD and UV/Vis spectra of D-RAO solutions with concentrations of 40, 55, 70, 85, 100 µM. c, d) absorbance vs concentration and CD vs absorbance plots at 200 nm and linear fits to these data. Slope of the line at panel d is used to calculate the ee of RAO solutions.

In order to calculate the ee of crystals we re-crystallized on magnetite we took CD calibration curves of enantiopure D-RAO. We prepared 5 solutions of D-RAO with varying concentrations at 40 μM, 55 μM, 70 μM, 85 μM, and 100 μM. We obtained the CD and UV-Vis spectra of these solutions. We then picked a wavelength to calibrate our samples. We picked 200 nm as the solvent and cuvette absorption is less at this wavelength compared to deeper UV wavelengths. Moreover, at this wavelength the slopes of the CD and UV-Vis curves are steeper, therefore the calibration is more sensitive at this wavelength compared to a plateau around 190 nm. Moreover, when we fitted the calibration lines (y = mx + c) to points taken at this wavelength, the constant term, c, was closer to 0 than other points. In an ideal scenario the constant term, c, should be 0 because if there is no absorption there should be no CD signal. Before we picked the points for fitting from the CD spectra, we smoothened the spectra using Savitzky-Golay filter with a window length of 101 and polynomial order of 3. Fig. S19a shows the raw CD spectra of 5 solutions of D-RAO at different concentrations and the smoothened spectra for each as the black lines. We then picked the CD angle and absorbance at 200 nm from the smoothened CD and raw UV-Vis spectra and plotted them against each other as shown in Fig. S19d. We used least squares fitting for the linear fits and obtained the following lines and fitting errors:

CD/Absorbance line at 200 nm: **(CD) = -19.187 (Abs) + 0.0375**

Standard error of the slope from the least-squares fit to the data: **0.590**

Standard error of the constant from the least-squares fit to the data: **0.168**

We also fitted UV-Vis absorbance at 200 nm to the pre-measured concentrations (Fig. S19c) to calculate the concentration of samples by using their UV-Vis absorbance at 200 nm. We used least squares fitting for the linear fits and obtained the following lines and fitting errors:

Abs/concentration line at 200 nm: **(Abs) = 0.0044 (Concentration in uM) – 0.0435**

Standard error of the slope from the least-squares fit to the data: **0.0002**

Standard error of the constant from the least-squares fit to the data: **0.012**

9. Enantiomeric excess calculations

We used the slope (m = -19.187) of the linear fit shown in Fig. S19d to calculate the enantiomeric excess using the following formula:

$$\%ee = \frac{\frac{CD\ angle\ at\ 200\ nm}{UV - Vis\ absorabnce\ at\ 200\ nm}}{m} \times 100$$

This formula calculates the ratio of the *absorbance normalized CD signal* of the sample and of enantiopure RAO. Due to the standard deviation of the linear fit 3% error in the ee is expected at

minimum. Due to additional errors introduced by smoothening and background absorption we assume an error of about +-5% in the calculated ee.

10. RAO crystallization with other aminoxazolines

10.1 General method for RAO crystallization with other aminooxazolines

We crystallized RAO with other aminooxazolines to understand the twinning properties of RAO crystal clusters and the effects of habit modifiers on crystallization. We prepared 2 mL solutions of RAO with other aminooxazolines and crystallized them in a Petri dish for a few hours and investigated the shapes of crystals.

10.2 RAO crystallization with XAO and habit modification

We crystallized 50 mM D-RAO with 50 mM D-AAO, D-XAO, and D-LAO separately. We observed that D-RAO forms needle shaped crystals in the presence of D-XAO and the other crystallizations gave regular diamond shaped crystals.

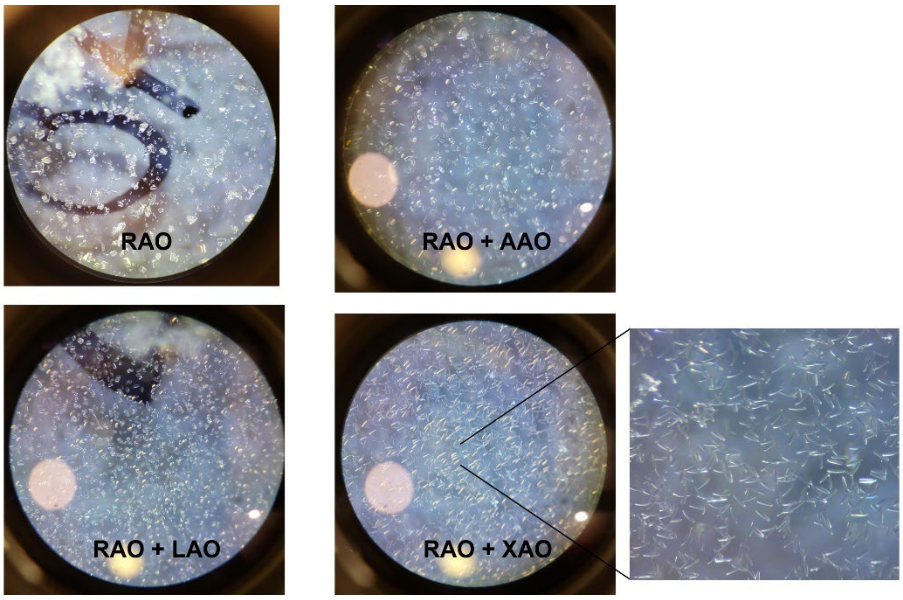

Fig. S20: 1 equivalent of D-RAO was crystallized with 1 equivalent of D-AAO, D-LAO, or D-XAO. We observed that in the presence of D-XAO, D-RAO forms crystals with different morphology. We used 50 mM concentration for all the crystallizations.

We then crystallized L-RAO with D-XAO (1:1, 50 mM) and did not observe the needle formation which indicates that the relative stereochemistry matters for the crystal habit modification. We also observed that D-XAO is embedded in the needle shaped crystals by ^{1}H NMR, as seen in Fig. S21. For this measurement, after the crystallization, we washed the crystals three times with pure water to make sure that

all supernatant was washed away and what we were measuring was only the dissolved crystals of RAO.

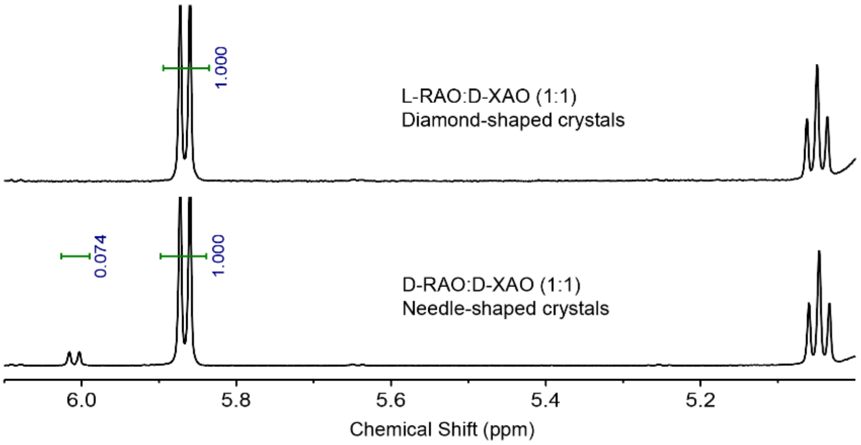

Fig. S21: Partial ^{1}H NMR spectra of the dissolved crystals of RAO. Top row corresponds to diamond-shaped crystals when L-RAO is crystallized with D-XAO. Bottom row corresponds to needle-shaped crystals when D-RAO is crystallized with D-XAO. For both we used 50 mM concentrations with 1:1 stoichiometry. As seen, D-XAO is embedded in the D-RAO crystals.

We have also observed that the morphology of RAO crystals changes when XAO is in the solution and the two aminooxazolines are in differing proportions. See Fig. S26.

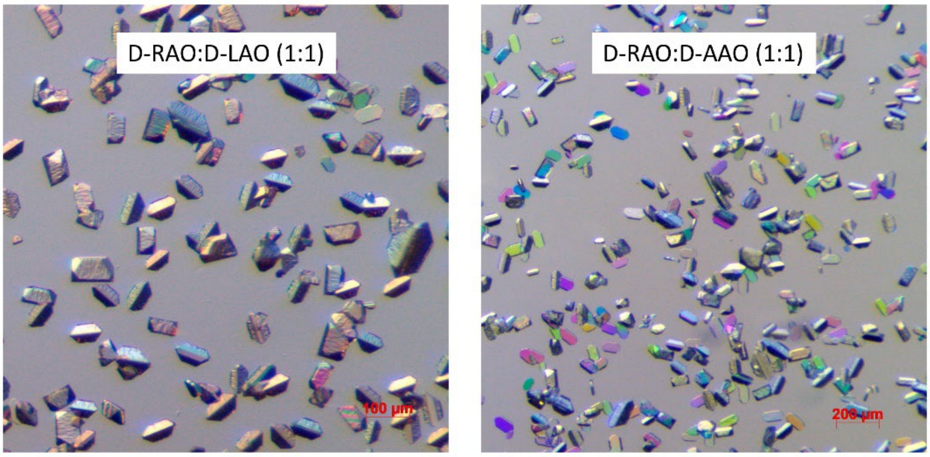

Fig. S22: When D-RAO is re-crystallized it forms diamond-shaped crystals. Similarly, when D-RAO is crystallized either with D-AAO or with D-LAO, diamond-shaped crystals are formed. For both experiments we used 50 mM D-RAO.

Last, at high concentrations we observed that in the presence of XAO, RAO forms twinned rosette-shaped clusters. We observed these clusters when we crystallized 250 mM of D-RAO in the presence of 62.5 mM D-XAO (4:1). We chose 4:1 as this mixing ratio is close to the one obtained when 2-aminooxazole adds to glyceraldehyde. We also observed that at high concentrations, these crystals form regardless of the ee of the solution. We observed rosette-shaped clusters when DL-RAO is crystallized with DL-XAO (4:1) as well.

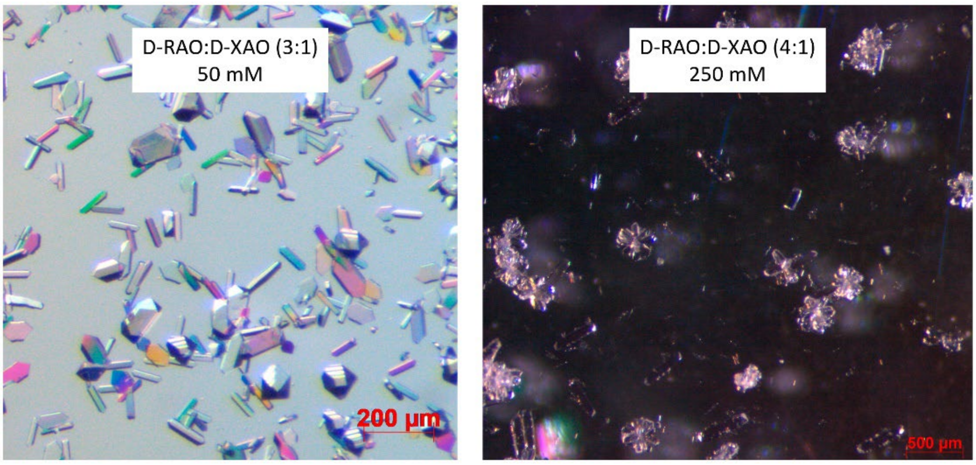

Fig. S23: At low concentrations (left panel), D-RAO forms elongated diamond-shaped crystals in the presence of D-XAO. However, at higher concentrations (right panel) these crystals aggregate into rosette-shaped crystal clusters.

10.3 Twinning properties of rosette-shaped RAO crystals

DL-RAO crystals form rosette-shaped twinned crystal clusters made from individually enantiopure needle-shaped crystals. We confirmed this by analyzing each needle-shaped arm of a cluster by X-ray diffraction. As seen on the right panel of Fig. S24, individual arms of a twinned cluster are enantiopure, yet the arrangement of the D and L crystals are randomly varying. Therefore, the whole cluster, if it consists of a non-stochastic number of needles, is on average racemic.

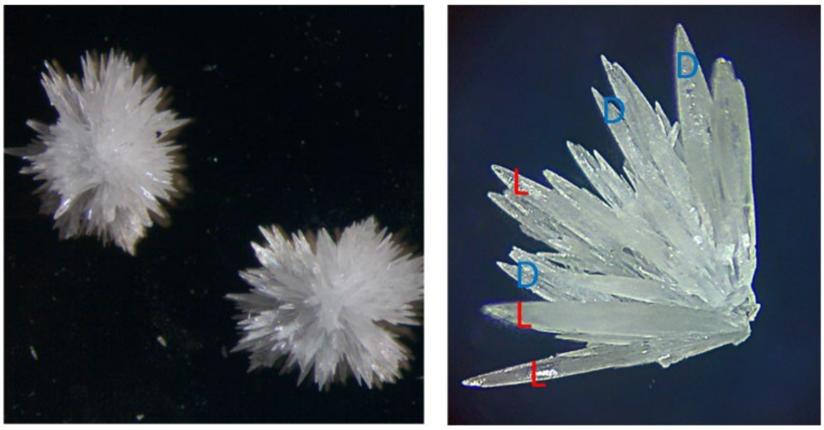

Fig. S24: Rosette-shaped crystal clusters of RAO. Individual needle-shaped arms of the cluster are enantiopure, yet the arrangement of the D and L needles is randomly varying, as confirmed by X-ray diffraction.

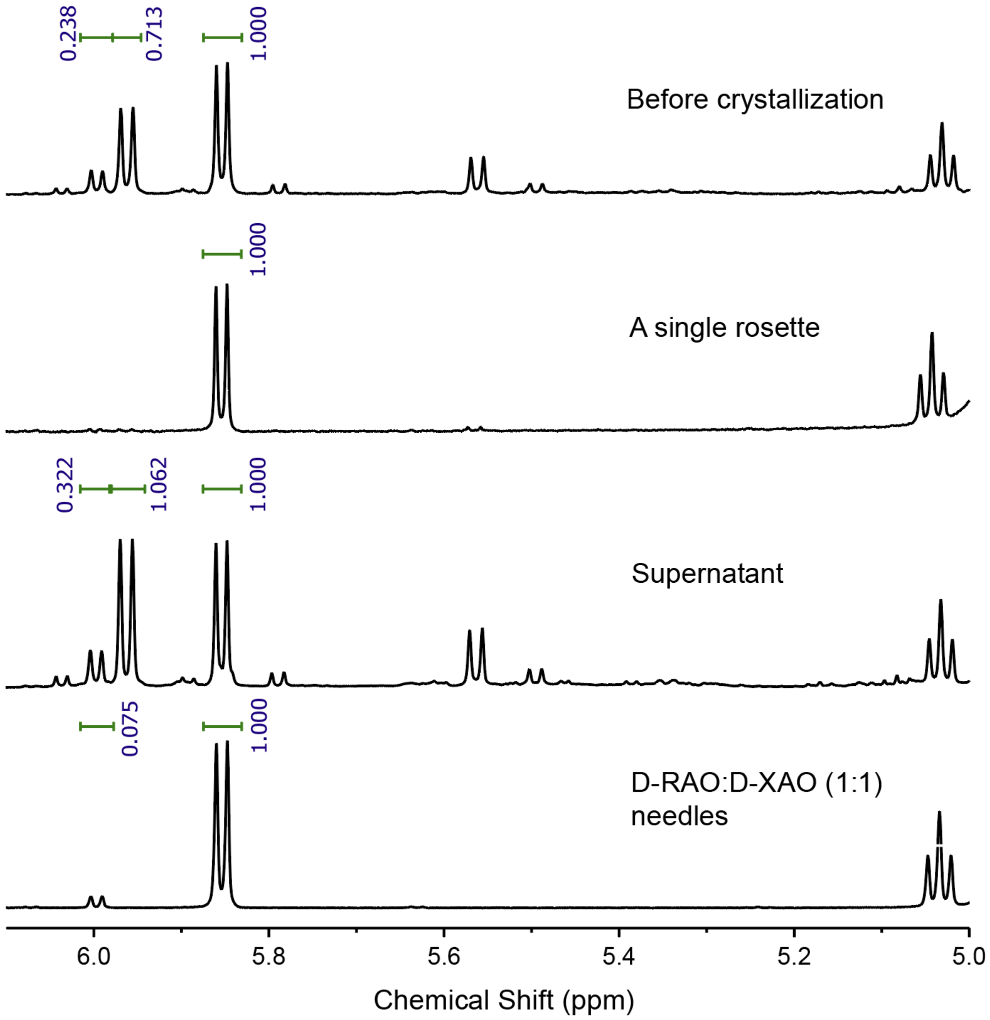

Fig. S25: ^{1}H NMR spectra of an individual rosette (second row) does not show XAO inclusion. Similarly, at prebiotic mixing ratios of RAO and XAO (about 4:1) we have not observed the presence of XAO in the crystals unlike the case with a 1:1 mixing ratio (fourth row). This is likely due to the limits of NMR detection. The supernatant (liquid above the rosette-shaped crystals, third row) is depleted in RAO after the crystallization compared to before (first row).

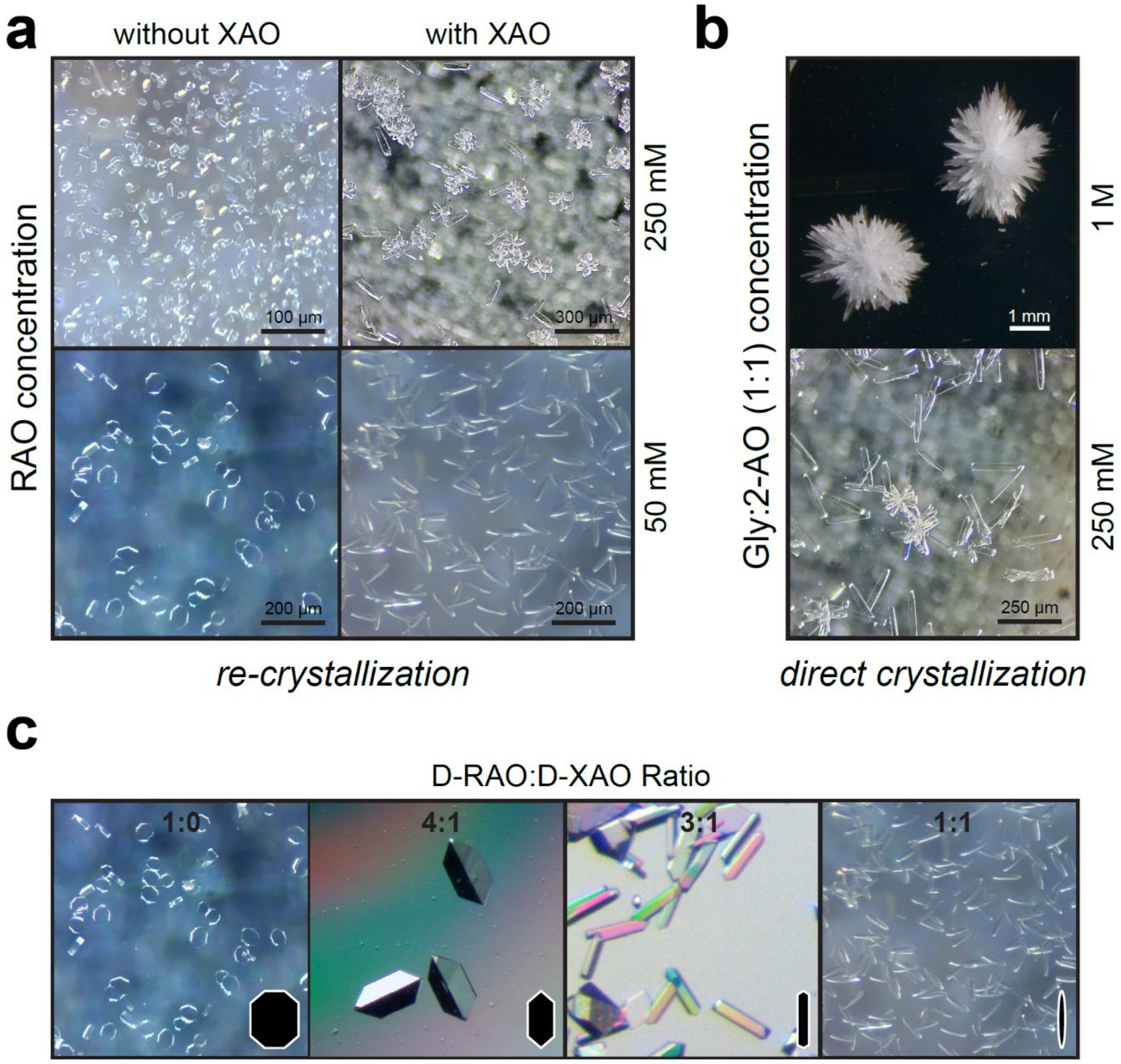

Fig. S26: Crystal habit modification of RAO by XAO a. RAO crystallization is modified with XAO. In the presence of XAO and at high concentrations, RAO forms rosette-shaped twinned conglomerates. **b.** Direct crystallization of RAO gives rosette-shaped twinned clusters, and as the concentration is decreased the conglomerate crystals become more angular and the rosette formation is suppressed. At lower concentrations for direct crystallization, the crystal morphology becomes identical to re-crystallized RAO in the presence of XAO. c. XAO modifies the crystal shape of RAO by inhibiting the growth of the side face and thus promoting the growth vertically. This makes the RAO crystals more elongated and eventually needle-shaped with increasing XAO abundance. On the top-center, the ratio between D-RAO and D-XAO; on the bottom-right, the sketch of the crystal shape is shown.

11. X-ray crystallography measurements

11.1 General method for X-ray crystallography measurements

A crystal mounted on a diffractometer and data was collected at 100 K. The intensities of the reflections were collected by means of a Bruker D8 Venture diffractometer ($Cu_{K\alpha}$ radiation, λ=1.54178 Å), and equipped with an Oxford Cryosystems nitrogen flow apparatus. The collection method involved 1.0° scans in ω at -68°, -24°, 24°, 68°, and 113° in 2θ. Data integration down to 0.84 Å resolution was carried out using SAINT V8.37A (*58*) with reflection spot size optimization. Absorption corrections were made with the program SADABS (*58*, *59*). The structure was solved by the Intrinsic Phasing methods and refined by least-squares methods again *F* (*59*) using SHELXT-2014 (*60*) and SHELXL-2014 (*60*) with OLEX 2 interface (*61*). Non-hydrogen atoms were refined anisotropically, and hydrogen atoms were allowed to ride on the respective atoms. Crystal data as well as details of data collection and refinement are summarized in Tables S3, S6, S9, geometric parameters are shown in Tables S4, S7, S10 and hydrogen-bond parameters are listed in Tables S5, S8, S11. The Ortep plots produced with SHELXL-2014 program, and the three-dimensional supramolecular architecture drawing was produced with Accelrys DS Visualizer 2.06 (*62*).

11.2 D-RAO

Experimental details

	D-RAO
Crystal data	
Chemical formula	$C_6H_{10}N_2O_4$
M_r	174.16
Crystal system, space group	Orthorhombic, $P2_12_12_1$
Temperature (K)	100
a, *b*, *c* (Å)	8.3674 (2), 8.5676 (2), 10.0411 (3)
V (Å^3)	719.83 (3)
Z	4
Radiation type	Cu $K\alpha$
μ (mm^{-1})	1.17
Crystal size (mm)	0.18 × 0.10 × 0.08
Data collection	
Diffractometer	Bruker D8 goniometer with Photon area detector
Absorption correction	Multi-scan<br>*SADABS*
T_{min}, T_{max}	0.681, 0.753

No. of measured, independent and observed [$I > 2\sigma(I)$] reflections	25337, 1270, 1261
R_{int}	0.030
$(\sin \theta/\lambda)_{max}$ (Å^{-1})	0.595
Refinement	
$R[F^2 > 2\sigma(F^2)]$, $wR(F^2)$, S	0.022, 0.058, 1.11
No. of reflections	1270
No. of parameters	126
H-atom treatment	H atoms treated by a mixture of independent and constrained refinement
$\Delta\rho_{max}$, $\Delta\rho_{min}$ (e Å^{-3})	0.21, -0.19
Absolute structure	Flack x determined using 506 quotients [(I+)-(I-)]/[(I+)+(I-)] (Parsons, Flack and Wagner, Acta Cryst. B69 (2013) 249-259).
Absolute structure parameter	0.04 (6)

Table S3: Experimental details of the D-RAO crystal measurements.

Computer programs: *SAINT* 8.37A (Bruker-AXS, 2015), *SHELXT2014* (Sheldrick, 2015), *SHELXL2014* (Sheldrick, 2015), Bruker *SHELXTL* (Sheldrick, 2015).

Geometric parameters (Å, º)

O1—C1	1.412 (2)	N2—C4	1.454 (2)
O1—H1	0.87 (3)	C1—C2	1.525 (2)
O2—C3	1.358 (2)	C1—C5	1.526 (2)
O2—C2	1.4548 (19)	C1—H1C	1.0000
O3—C4	1.445 (2)	C2—C4	1.541 (3)
O3—C5	1.446 (2)	C2—H2	1.0000
O4—C6	1.425 (2)	C4—H4A	1.0000
O4—H4	0.86 (3)	C5—C6	1.511 (2)
N1—C3	1.336 (2)	C5—H5	1.0000
N1—H1A	0.89 (3)	C6—H6A	0.9900
N1—H1B	0.85 (3)	C6—H6B	0.9900
N2—C3	1.292 (2)		
C1—O1—H1	108.4 (16)	N2—C3—O2	118.18 (16)
C3—O2—C2	105.96 (13)	N1—C3—O2	113.86 (16)
C4—O3—C5	107.27 (13)	O3—C4—N2	111.75 (14)
C6—O4—H4	109.8 (18)	O3—C4—C2	105.62 (14)

C3—N1—H1A	118.5 (16)	N2—C4—C2	105.36 (13)
C3—N1—H1B	116.7 (16)	O3—C4—H4A	111.3
H1A—N1—H1B	120 (2)	N2—C4—H4A	111.3
C3—N2—C4	106.58 (15)	C2—C4—H4A	111.3
O1—C1—C2	112.15 (14)	O3—C5—C6	110.26 (14)
O1—C1—C5	113.34 (14)	O3—C5—C1	101.80 (13)
C2—C1—C5	102.43 (14)	C6—C5—C1	116.35 (15)
O1—C1—H1C	109.6	O3—C5—H5	109.4
C2—C1—H1C	109.6	C6—C5—H5	109.4
C5—C1—H1C	109.6	C1—C5—H5	109.4
O2—C2—C1	110.17 (13)	O4—C6—C5	112.96 (14)
O2—C2—C4	103.73 (13)	O4—C6—H6A	109.0
C1—C2—C4	104.23 (13)	C5—C6—H6A	109.0
O2—C2—H2	112.7	O4—C6—H6B	109.0
C1—C2—H2	112.7	C5—C6—H6B	109.0
C4—C2—H2	112.7	H6A—C6—H6B	107.8
N2—C3—N1	127.95 (17)		
C3—O2—C2—C1	113.85 (16)	C3—N2—C4—C2	4.20 (18)
C3—O2—C2—C4	2.79 (17)	O2—C2—C4—O3	114.17 (13)
O1—C1—C2—O2	36.69 (19)	C1—C2—C4—O3	-1.19 (17)
C5—C1—C2—O2	-85.16 (15)	O2—C2—C4—N2	-4.24 (17)
O1—C1—C2—C4	147.42 (14)	C1—C2—C4—N2	-119.59 (14)
C5—C1—C2—C4	25.57 (17)	C4—O3—C5—C6	166.13 (14)
C4—N2—C3—N1	176.76 (18)	C4—O3—C5—C1	42.05 (16)
C4—N2—C3—O2	-2.7 (2)	O1—C1—C5—O3	-162.05 (13)
C2—O2—C3—N2	-0.2 (2)	C2—C1—C5—O3	-41.01 (16)
C2—O2—C3—N1	-179.73 (14)	O1—C1—C5—C6	78.07 (19)
C5—O3—C4—N2	88.36 (16)	C2—C1—C5—C6	-160.89 (14)
C5—O3—C4—C2	-25.70 (16)	O3—C5—C6—O4	-59.37 (19)
C3—N2—C4—O3	-110.01 (15)	C1—C5—C6—O4	55.8 (2)

Table S4: Geometric parameters of the D-RAO crystal.

Hydrogen-bond parameters

D—H···*A*	*D*—H (Å)	H···*A* (Å)	*D*···*A* (Å)	*D*—H···*A* (°)
O1—H1···O3^{i}	0.87 (3)	1.82 (3)	2.6759 (17)	168 (2)
O4—H4···N2^{i}	0.86 (3)	1.93 (3)	2.778 (2)	174 (3)
N1—H1B···O1^{ii}	0.85 (3)	2.28 (2)	2.862 (2)	126 (2)
N1—H1A···O4^{iii}	0.89 (3)	2.00 (3)	2.892 (2)	177 (2)

Table S5: Hydrogen-bond parameters of the D-RAO crystal.

Symmetry code(s): (i) -*x*-3/2, -*y*-1, *z*+1/2; (ii) -*x*-3/2, -*y*-2, *z*-1/2; (iii) *x*, *y*-1, *z*.

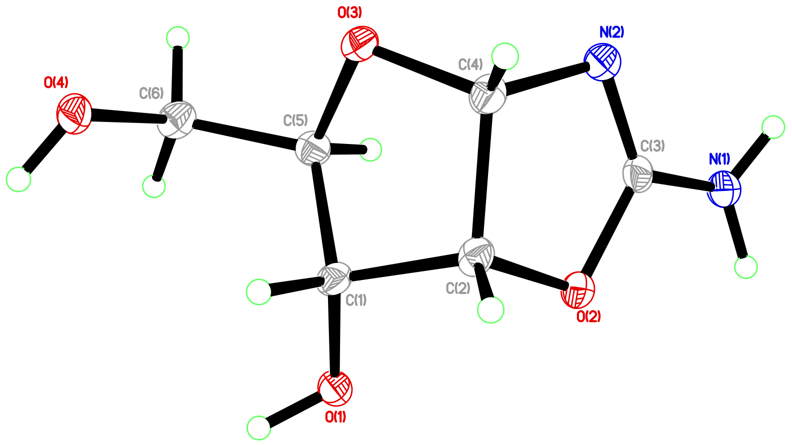

Fig. S27. Perspective views showing 50% probability displacement for D-RAO.

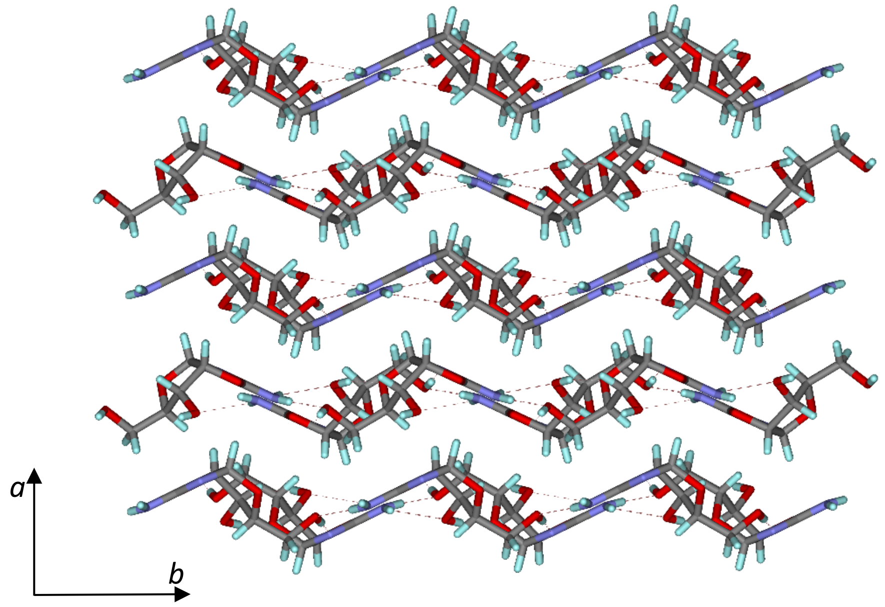

Fig. S28. Three-dimensional supramolecular architecture viewed along the *c*-axis direction.

11.3 L-RAO

Experimental details

	L-RAO
Crystal data	
Chemical formula	$C_6H_{10}N_2O_4$
M_r	174.16
Crystal system, space group	Orthorhombic, $P2_12_12_1$
Temperature (K)	100
a, *b*, *c* (Å)	8.3646 (2), 8.5700 (2), 10.0383 (2)
V (Å^3)	719.59 (3)
Z	4
Radiation type	Cu $K\alpha$
μ (mm^{-1})	1.17
Crystal size (mm)	0.18 × 0.12 × 0.10
Data collection	
Diffractometer	Bruker D8 goniometer with Photon area detector
Absorption correction	Multi-scan<br>*SADABS*
T_{min}, T_{max}	0.757, 0.806
No. of measured, independent and observed [$I > 2\sigma(I)$] reflections	13526, 1264, 1250
R_{int}	0.032
$(\sin \theta/\lambda)_{max}$ (Å^{-1})	0.595
Refinement	
$R[F^2 > 2\sigma(F^2)]$, $wR(F^2)$, S	0.022, 0.057, 1.09
No. of reflections	1264
No. of parameters	126
H-atom treatment	H atoms treated by a mixture of independent and constrained refinement
$\Delta\rho_{max}$, $\Delta\rho_{min}$ (e Å^{-3})	0.19, -0.14
Absolute structure	Flack x determined using 499 quotients [(I+)-(I-)]/[(I+)+(I-)] (Parsons, Flack and Wagner, Acta Cryst. B69 (2013) 249-259).
Absolute structure parameter	-0.01 (6)

Table S6: Experimental details of the L-RAO crystal measurements.

Geometric parameters (Å, º)

O1—C1	1.413 (2)	N2—C4	1.454 (2)
O1—H1	0.87 (2)	C1—C5	1.525 (2)
O2—C3	1.356 (2)	C1—C2	1.526 (2)
O2—C2	1.4554 (19)	C1—H1C	1.0000
O3—C4	1.444 (2)	C2—C4	1.540 (3)
O3—C5	1.446 (2)	C2—H2	1.0000
O4—C6	1.425 (2)	C4—H4A	1.0000
O4—H4	0.85 (3)	C5—C6	1.512 (2)
N1—C3	1.336 (2)	C5—H5	1.0000
N1—H1A	0.87 (3)	C6—H6A	0.9900
N1—H1B	0.86 (2)	C6—H6B	0.9900
N2—C3	1.292 (2)		
C1—O1—H1	110.1 (16)	N2—C3—O2	118.15 (16)
C3—O2—C2	105.99 (13)	N1—C3—O2	113.99 (16)
C4—O3—C5	107.26 (13)	O3—C4—N2	111.76 (14)
C6—O4—H4	110.1 (17)	O3—C4—C2	105.65 (14)
C3—N1—H1A	119.1 (16)	N2—C4—C2	105.36 (13)
C3—N1—H1B	117.5 (15)	O3—C4—H4A	111.3
H1A—N1—H1B	119 (2)	N2—C4—H4A	111.3
C3—N2—C4	106.59 (14)	C2—C4—H4A	111.3
O1—C1—C5	113.34 (14)	O3—C5—C6	110.23 (14)
O1—C1—C2	112.12 (14)	O3—C5—C1	101.77 (13)
C5—C1—C2	102.39 (14)	C6—C5—C1	116.29 (15)
O1—C1—H1C	109.6	O3—C5—H5	109.4
C5—C1—H1C	109.6	C6—C5—H5	109.4
C2—C1—H1C	109.6	C1—C5—H5	109.4
O2—C2—C1	110.18 (13)	O4—C6—C5	112.98 (14)
O2—C2—C4	103.71 (13)	O4—C6—H6A	109.0
C1—C2—C4	104.21 (13)	C5—C6—H6A	109.0
O2—C2—H2	112.7	O4—C6—H6B	109.0
C1—C2—H2	112.7	C5—C6—H6B	109.0
C4—C2—H2	112.7	H6A—C6—H6B	107.8
N2—C3—N1	127.85 (17)		

C3—O2—C2—C1	-113.90 (15)	C3—N2—C4—C2	-4.16 (19)
C3—O2—C2—C4	-2.87 (17)	O2—C2—C4—O3	-114.17 (14)
O1—C1—C2—O2	-36.71 (19)	C1—C2—C4—O3	1.17 (17)
C5—C1—C2—O2	85.10 (15)	O2—C2—C4—N2	4.26 (17)
O1—C1—C2—C4	-147.41 (14)	C1—C2—C4—N2	119.60 (14)
C5—C1—C2—C4	-25.60 (17)	C4—O3—C5—C6	-166.11 (14)
C4—N2—C3—N1	-176.68 (18)	C4—O3—C5—C1	-42.14 (16)
C4—N2—C3—O2	2.6 (2)	O1—C1—C5—O3	162.04 (13)
C2—O2—C3—N2	0.3 (2)	C2—C1—C5—O3	41.07 (16)
C2—O2—C3—N1	179.69 (14)	O1—C1—C5—C6	-78.17 (19)
C5—O3—C4—N2	-88.31 (16)	C2—C1—C5—C6	160.86 (14)
C5—O3—C4—C2	25.76 (16)	O3—C5—C6—O4	59.34 (19)
C3—N2—C4—O3	110.09 (16)	C1—C5—C6—O4	-55.8 (2)

Table S7: Geometric parameters of the L-RAO crystal.

Hydrogen-bond parameters

D—H···*A*	*D*—H (Å)	H···*A* (Å)	*D*···*A* (Å)	*D*—H···*A* (°)
O1—H1···O3^{i}	0.87 (2)	1.83 (3)	2.6762 (17)	165 (2)
O4—H4···N2^{i}	0.85 (3)	1.94 (3)	2.7789 (19)	173 (2)
N1—H1B···O1^{ii}	0.86 (2)	2.27 (2)	2.8626 (19)	126.7 (18)
N1—H1A···O4^{iii}	0.87 (3)	2.03 (3)	2.893 (2)	177 (2)

Table S8: Hydrogen-bond parameters of the L-RAO crystal.

Symmetry code(s): (i) -*x*+3/2, -*y*+1, *z*-1/2; (ii) -*x*+3/2, -*y*+2, *z*+1/2; (iii) *x*, *y*+1, *z*.

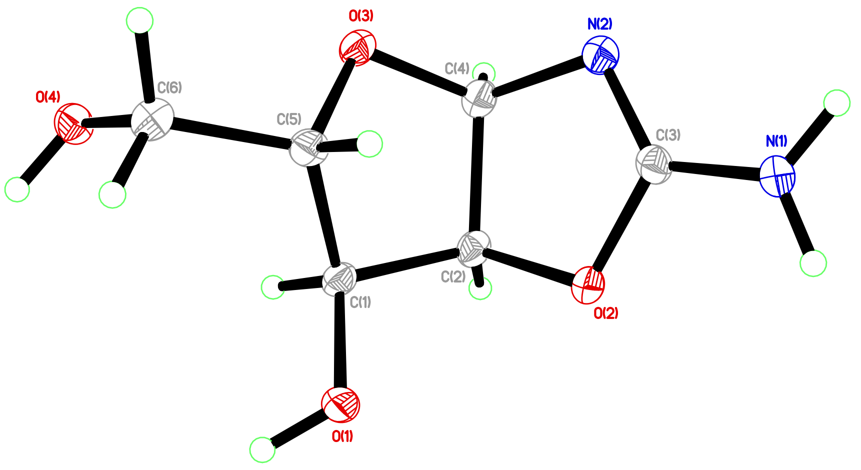

Fig. S29. Perspective views showing 50% probability displacement for L-RAO.

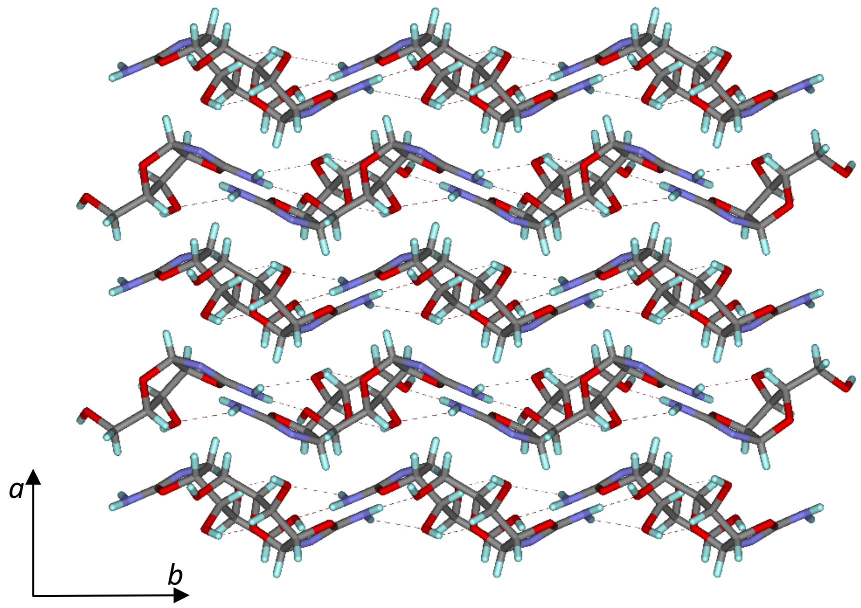

Fig. S30. Three-dimensional supramolecular architecture viewed along the *c*-axis direction.

11.4 D-RAO and D-XAO

Experimental details

	D-RAO+XAO
Crystal data	
Chemical formula	$C_6H_{10}N_2O_4$
M_r	174.16
Crystal system, space group	Orthorhombic, $P2_12_12_1$
Temperature (K)	100
a, *b*, *c* (Å)	8.3776 (16), 8.5683 (15), 10.0666 (17)
V (Å^3)	722.6 (2)
Z	4
Radiation type	Cu $K\alpha$
μ (mm^{-1})	1.17
Crystal size (mm)	0.18 × 0.10 × 0.08
Data collection	
Diffractometer	Bruker D8 goniometer with Photon area detector
Absorption correction	Multi-scan<br>*SADABS*
T_{min}, T_{max}	0.754, 0.806
No. of measured, independent and observed [$I > 2\sigma(I)$] reflections	9593, 1270, 1208
R_{int}	0.048
$(\sin \theta/\lambda)_{max}$ (Å^{-1})	0.595
Refinement	
$R[F^2 > 2\sigma(F^2)]$, $wR(F^2)$, S	0.031, 0.067, 1.10
No. of reflections	1270
No. of parameters	129
No. of restraints	1
H-atom treatment	H atoms treated by a mixture of independent and constrained refinement
$\Delta\rho_{max}$, $\Delta\rho_{min}$ (e Å^{-3})	0.16, -0.18
Absolute structure	Flack x determined using 459 quotients [(I+)-(I-)]/[(I+)+(I-)] (Parsons, Flack and Wagner, Acta Cryst. B69 (2013) 249-259).
Absolute structure parameter	-0.21 (16)

Table S9: Experimental details of the D-RAO:D-XAO crystal measurements.

Geometric parameters (Å, º)

O1—C1	1.412 (3)	N2—C3	1.294 (3)
O1—H1	0.89 (4)	N2—C4	1.457 (4)
O1A—C1	1.40 (2)	C1—C2	1.523 (4)
O1A—H1A	0.9825	C1—C5	1.526 (3)
O2—C3	1.359 (3)	C1—H1AA	1.0000
O2—C2	1.459 (3)	C1—H1BB	1.0000
O3—C5	1.444 (3)	C2—C4	1.540 (4)
O3—C4	1.447 (3)	C2—H2	1.0000
O4—C6	1.431 (3)	C4—H4A	1.0000
O4—H4	0.87 (3)	C5—C6	1.512 (3)
N1—C3	1.339 (4)	C5—H5	1.0000
N1—H1B	0.90 (4)	C6—H6A	0.9900
N1—H1C	0.84 (4)	C6—H6B	0.9900
C1—O1—H1	110 (3)	C1—C2—H2	112.7
C1—O1A—H1A	113.9	C4—C2—H2	112.7
C3—O2—C2	105.95 (18)	N2—C3—N1	127.9 (2)
C5—O3—C4	107.18 (18)	N2—C3—O2	118.3 (2)
C6—O4—H4	110 (2)	N1—C3—O2	113.8 (2)
C3—N1—H1B	118 (2)	O3—C4—N2	111.7 (2)
C3—N1—H1C	117 (2)	O3—C4—C2	105.6 (2)
H1B—N1—H1C	121 (3)	N2—C4—C2	105.6 (2)
C3—N2—C4	106.4 (2)	O3—C4—H4A	111.2
O1A—C1—C2	106.4 (17)	N2—C4—H4A	111.2
O1—C1—C2	112.3 (2)	C2—C4—H4A	111.2
O1A—C1—C5	114.8 (15)	O3—C5—C6	110.2 (2)
O1—C1—C5	113.4 (2)	O3—C5—C1	101.90 (19)
C2—C1—C5	102.3 (2)	C6—C5—C1	116.6 (2)
O1—C1—H1AA	109.5	O3—C5—H5	109.2
C2—C1—H1AA	109.5	C6—C5—H5	109.2
C5—C1—H1AA	109.5	C1—C5—H5	109.2
O1A—C1—H1BB	110.9	O4—C6—C5	112.9 (2)
C2—C1—H1BB	110.9	O4—C6—H6A	109.0
C5—C1—H1BB	110.9	C5—C6—H6A	109.0
O2—C2—C1	110.25 (19)	O4—C6—H6B	109.0

O2—C2—C4	103.61 (19)	C5—C6—H6B	109.0
C1—C2—C4	104.36 (19)	H6A—C6—H6B	107.8
O2—C2—H2	112.7		
C3—O2—C2—C1	114.1 (2)	C3—N2—C4—C2	4.1 (3)
C3—O2—C2—C4	2.9 (2)	O2—C2—C4—O3	114.2 (2)
O1A—C1—C2—O2	154.1 (15)	C1—C2—C4—O3	-1.2 (2)
O1—C1—C2—O2	36.8 (3)	O2—C2—C4—N2	-4.3 (3)
C5—C1—C2—O2	-85.1 (2)	C1—C2—C4—N2	-119.7 (2)
O1A—C1—C2—C4	-95.3 (15)	C4—O3—C5—C6	166.5 (2)
O1—C1—C2—C4	147.5 (2)	C4—O3—C5—C1	42.1 (2)
C5—C1—C2—C4	25.6 (2)	O1A—C1—C5—O3	73.8 (18)
C4—N2—C3—N1	176.7 (3)	O1—C1—C5—O3	-162.21 (19)
C4—N2—C3—O2	-2.5 (3)	C2—C1—C5—O3	-41.1 (2)
C2—O2—C3—N2	-0.4 (3)	O1A—C1—C5—C6	-46.3 (18)
C2—O2—C3—N1	-179.7 (2)	O1—C1—C5—C6	77.7 (3)
C5—O3—C4—N2	88.6 (2)	C2—C1—C5—C6	-161.2 (2)
C5—O3—C4—C2	-25.7 (2)	O3—C5—C6—O4	-59.5 (3)
C3—N2—C4—O3	-110.1 (2)	C1—C5—C6—O4	56.0 (3)

Table S10: Geometric parameters of the D-RAO:D-XAO crystal.

Hydrogen-bond parameters

D—H···*A*	*D*—H (Å)	H···*A* (Å)	*D*···*A* (Å)	*D*—H···*A* (°)
O1—H1···O3^{i}	0.89 (4)	1.81 (4)	2.680 (3)	166 (4)
O1A—H1A···N2^{ii}	0.98	2.43	2.86 (3)	106.1
O4—H4···N2^{i}	0.87 (3)	1.91 (3)	2.778 (3)	175 (3)
N1—H1C···O1^{iii}	0.84 (4)	2.29 (3)	2.859 (3)	125 (3)
N1—H1B···O4^{iv}	0.90 (4)	1.99 (4)	2.889 (3)	176 (3)

Table S11: Hydrogen-bond parameters of the D-RAO:D-XAO crystal.

Symmetry code(s): (i) -*x*+1/2, -*y*+1, *z*+1/2; (ii) -*x*+1, *y*+1/2, -*z*+3/2; (iii) -*x*+1/2, -*y*, *z*-1/2; (iv) *x*, *y*-1, *z*.

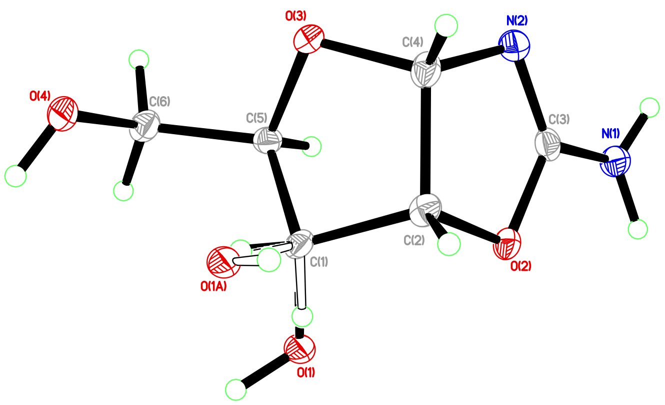

Fig. S31. Perspective views showing 50% probability displacement for a disordered model for mixture of D-RAO [95.2(4)%] and D-XAO [4.8(4)%].

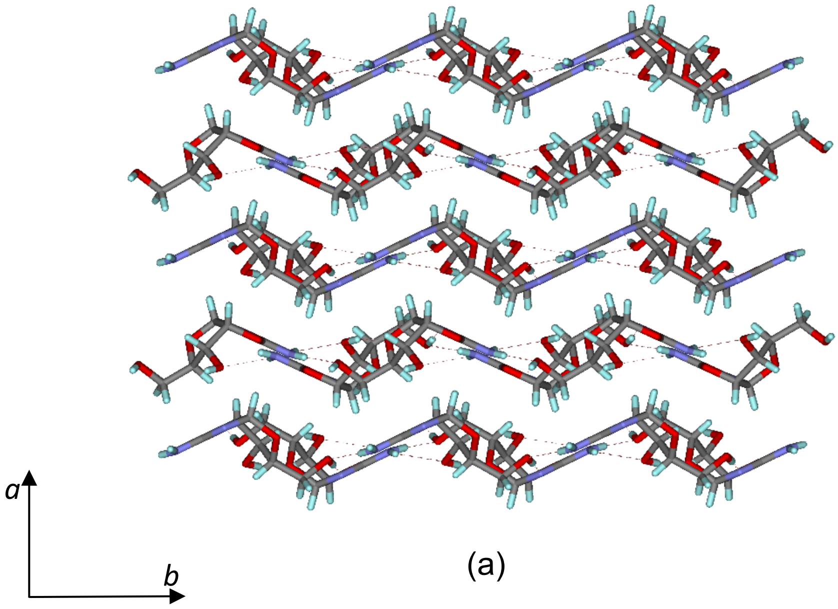

(a)

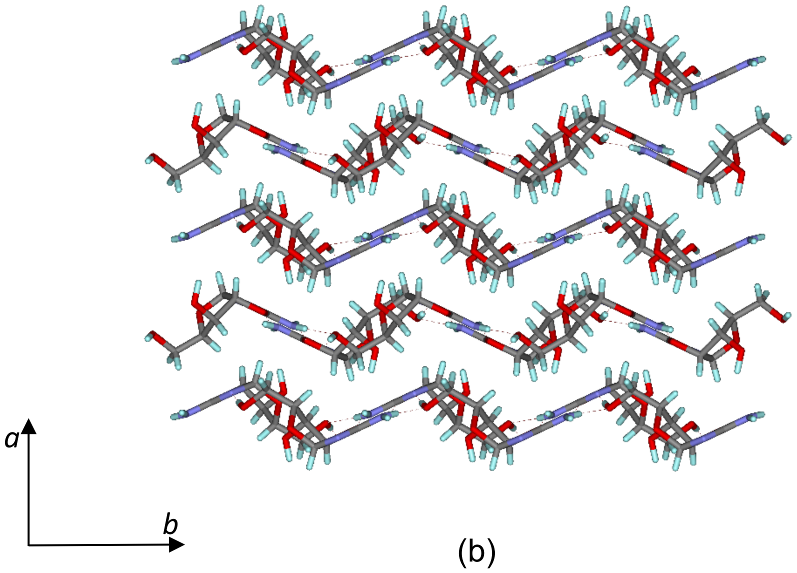

(b)

Fig. S32. Three-dimensional supramolecular architecture viewed along the *c*-axis direction for (a) D-RAO only and (b) D-XAO only.

Movie S1. A 24-hour-timelapse of direct crystallization of RAO from the reaction products of 1:1M DL-glyceraldehyde:2-AO on a magnetite surface. Crystallization is seeded by the magnetic surface in a spin-selective manner. However, due to the stochastic arrangement of homochiral domains in a rosette-shaped cluster, the crystal overall is not enantiopure.

Crystallographic Information Files

Crystallographic Information Files (CIF) representing the crystallographic properties of the crystals we obtained are attached as supplementary data.

Data S1. CIF file for D-RAO.

Data S2. CIF file for L-RAO.

Data S3. CIF file for D-RAO/D-XAO.
